# Tuning Nanoscale Conductance in Cyclic Molecules via Molecular Length and Anchoring Groups

**DOI:** 10.3390/nano16020083

**Published:** 2026-01-07

**Authors:** Abdullah Alshehab, Turki Alotaibi, Ali K. Ismael

**Affiliations:** 1 Department of Physics, College of Science, King Faisal University, Al Ahsa 31982, Saudi Arabia; 2 Department of Physics, College of Science, Jouf University, Sakaka P.O. Box 2014, Saudi Arabia; tbotaibi@ju.edu.sa; 3 Department of Physics, Lancaster University, Lancaster LA1 4YB, UK; 4 Department of Physics, College of Education for Pure Science, Tikrit University, Tikrit 34001, Iraq

**Keywords:** cyclic molecules, molecular conductance, anchor group

## Abstract

This theoretical study investigates the electrical conductance of non-conjugated cyclic molecules featuring three terminal anchoring groups at the single-molecule level. Density Functional Theory (DFT) calculations demonstrate that the conductance of the symmetric and asymmetric cyclic structures *C*_6_*C*_6_, *C*_6_*C*_8_, *C*_6_*C*_10_, *C*_8_*C*_8_, *C*_8_*C*_10_, and *C*_10_*C*_10_ (where the numbers indicate the lengths of the upper and lower branches) decreases with increasing molecular length, independent of the anchor group chemistry. Distinct trends are observed across molecular series: the 6-unit branch—defined as molecules containing a common six-carbon saturated segment (e.g., *C*_4_*C*_6_, *C*_6_*C*_6_, *C*_6_*C*_8_, *C*_6_*C*_10_)—exhibits a non-conventional pattern, whereas the 8-unit and 10-unit branches display parabolic and conventional length-dependent behavior, respectively. A key finding is that cyclic molecules with identical total CH_2_ units exhibit nearly identical conductance values, irrespective of structural symmetry. These theoretical predictions are strongly supported by previously reported scanning tunneling microscopy break-junction measurements, establishing a fundamental structure–property relationship for sigma-conjugated molecular systems. These findings provide critical design principles for developing advanced molecular-scale electronic devices.

## 1. Introduction

Research on electron transport through molecular junctions has matured into a sophisticated field, revealing a rich interplay of quantum phenomena and molecular structures. Theoretically, transport is primarily understood through two core mechanisms: coherent tunneling, where electrons traverse the molecule as a wave without energy loss, and sequential hopping, where electrons localize on molecular orbitals, assisted by thermal energy. Experimentally, techniques like scanning tunneling microscope break junctions (STM-BJs) and mechanically controllable break junctions (MCBJs) have been pivotal, enabling the statistical measurement of conductance across single molecules. A critical finding is that the conductance is not solely an intrinsic molecular property but is profoundly influenced by the alignment of molecular orbitals with the electrode’s Fermi level and the nature of the molecule–electrode contact interface. This synergy between theoretical modeling and advanced experimentation continues to drive the field, opening avenues for molecular electronics, quantum interference devices, and molecular-scale sensors [1]. In the future, a transport system like this will be critical to the development of electronics and may be used for various purposes. The past few decades have witnessed intensive research into various organic and inorganic molecules [2,3,4,5,6,7,8]. The application of molecular electronics has led to the observation of several significant phenomena, such as switching, rectification, and the detection of negative differential resistance. As a result, there is considerable potential in this field and a bright future ahead [9,10,11,12,13,14,15,16,17]. Scientists dream of using molecular devices to construct molecular computers, where traditional electronic components would be replaced by these devices. The research on molecular devices, however, poses several experimental and theoretical challenges [1]. Molecular conductance can be understood through a combination of theoretical calculations and experimental investigations of molecular devices based on well-defined structural properties of nanoscale molecules [18,19,20,21,22,23].

According to Kirchhoff’s law of superposition in classical physics, connecting two identical conductors in parallel results in double the conductance of a single conductor, producing a conventional trend. Parallel connections between two conductors within a molecular circuit, however, do not often produce conductance that is subject to the classical superposition law at the nanoscale [17]. The reason for this is that electron tunneling through a molecular circuit is governed by quantum physics principles rather than classical physics principles. At the nanoscale level, a theoretical study [24] supported by experimental measurements attempted to determine whether Kirchhoff’s law remains valid when applied to Sigma non-conjugated molecules. In their study, they demonstrated that Kirchhoff’s law does not apply to cyclic symmetric and asymmetric alkane molecules with different terminal end groups.

The study of Sigma systems of multi-branch molecules has gained increasing attention over the past few years. For instance, an early theoretical and experimental study concluded that the electrical conductance of alkane cyclic molecules decreases with an increase in ring diameter [25]. In recent years, theoretical and experimental work has been conducted on charge transport via Sigma systems with alkane cyclic molecules.

According to [20], heterocyclic alkanes have a higher conductance than their corresponding linear alkane chains. In this study, the STM-BJ method and calculations based on Density Functional Theory (DFT) demonstrated that three heterocyclic alkane rings, piperazine, *C*_222_-diaza, and dithiane, had lower conductance values than their equivalent linear alkanes. Therefore, organic molecules with destructive quantum interference can be utilized to design short molecular insulators. In heterocyclic alkanes, the absence of π-conjugated pathways combined with the limited efficiency of σ-mediated transmission generally results in low electrical conductance.

In a number of theoretical and experimental investigations, it has been demonstrated that an increase in molecular length has an important effect on the conductance at molecular junctions [26,27,28,29]. A recent experimental study [30] was carried out on the conductance of single molecules of [n]staffanes, where n represents the number of repeating units known as oligomers. This study aims to gain a better understanding of the length-dependent conductance properties of [n]staffanes. According to their findings, the conductance through [n]staffanes decays exponentially as the length of the oligomer increases. As an example, the most probable conductance value for a Staffane with three repeating units (3) was approximately 8.17 × 10^−4^ G_0_, while a Staffane with four repeating units had an approximate conductance value of 1 × 10^−6^ G_0_. The results of their DFT calculations are in agreement with their findings.

The investigation of electrical conductance in cyclic molecules is essential not only because their multiple intramolecular transport pathways can give rise to non-classical charge-transport behavior, but also because cyclic structures of different sizes can selectively bind analyte molecules, offering potential functionality for molecular sensing applications [25].

The electrical conductance of single-molecule junctions is primarily governed by the chemical identity of the anchoring groups that connect the molecule to metallic electrodes, as these groups determine both the strength of electronic coupling at the molecule–electrode interface and the alignment of the molecular frontier orbitals relative to the electrode Fermi level. Extensive experimental and theoretical studies have demonstrated that variations in terminal anchoring groups—including thiol (–SH), amine (–NH_2_), pyridyl (–Py), cyano (–CN), thiomethyl (–SMe), and direct carbon (–C)—result in pronounced differences in conductance arising from changes in binding geometry, coupling strength, and energy level alignment [31,32,33,34]. For linear alkane chains, for example, calculated conductance values follow the trend $G_{C}>G_{SH}>G_{SMe}>G_{{\mathrm{NH}}_{2}}$, reflecting progressively weaker molecule–electrode interactions from direct carbon to thiol, thiomethyl, and amine anchoring groups. These trends are in strong agreement with experimentally measured scanning tunneling microscopy break-junction (STM-BJ) conductance values, supporting the reliability of density functional theory (DFT) predictions [29]. Further insight from DFT combined with non-equilibrium Green’s function (NEGF) calculations indicates that stronger molecule–electrode coupling enhances electronic transmission near the Fermi level and effectively lowers the tunneling barrier [26,35]. Single-molecule conductance measurements using both STM-BJ and mechanically controllable break-junction (MCBJ) techniques further confirm that anchoring groups significantly influence junction formation probability and conductance distributions [31,32,33]. However, this anchoring-group trend is not universal and can differ markedly for cyclic molecular systems, where molecular topology and σ-pathway interference play a central role in governing charge transport. In such systems, anchoring-group effects must be interpreted in conjunction with molecular architecture and transport pathways, as evidenced by the conductance trends observed in the present study.

Building on established principles, this density functional theory (DFT) investigation systematically evaluates the influence of anchoring chemistry on charge transport in cyclic molecular junctions. By examining symmetric and asymmetric organic cyclic molecules terminated with amine, thiol, and direct carbon contacts, the study assesses how variations in molecule–electrode coupling affect the observed conductance (*G*). This model framework enables a direct comparison of anchor-dependent transport behavior in σ-mediated systems and demonstrates that anchoring groups are central to modulating electron transmission across cyclic molecules.

## 2. Methods

The first steps were to model the terminal group–Au binding and then to relax each molecule in the presence of two fixed leads. In this study, Density Functional Theory (DFT) and the SIESTA code [36,37,38,39,40] were used; we have determined the optimal geometries for isolated cyclic molecules by relaxing them until the atoms were subjected to force levels less than 0.05 eV/Å and one k-point (see Supplementary Tables S1–S6). Furthermore, we computed the results using LDA and found that they were similar to those obtained by GGA [41,42,43,44,45,46]. Gollum [47] is a quantum transport code used to calculate the electrical conductance after each molecular junction has been relaxed (more information can be found in Sections S3 and S4 of the Supplementary Materials).

In this DFT theoretical investigation, 51 molecules with three different anchor groups were examined. As shown in Table 1, some cyclic molecules contain direct carbon terminal groups. Two types of symmetric cyclic molecules exist: odd–odd and even–even (first and second columns, respectively). Asymmetric cyclic molecules are represented by odd–odd + 2 and even–even + 2 molecules (respectively, the third and fourth columns) (relaxed cyclic molecules with thiol and amine terminal groups can be found in Section S1 and Supplementary Tables S1–S6).

Throughout this study, all cyclic molecules were connected to gold electrodes with three terminal end groups, amines (Au-NH2), thiols (Au-S), and covalent bonds, to establish direct contact with carbon (Au-C). Some examples of optimized DFT structures of cyclic rings at their junctions are illustrated in Figure S2 of the Supplementary Materials.

We assigned two integers, *n* and *m*, to represent the branch lengths of the number of methylene CH_2_ units as an efficient way of discussing the conductance of cyclic molecules with two branches. Using this approach, we constructed a series of *CnCm* cyclic molecules with two branches, *n* and *m*, whose ranges could be determined by the formula 3≤n,m≤12 with three different anchor groups. Considering that we studied many molecules, we divided this study into symmetric and asymmetric cyclic alkane molecules. The CH_2_ units in *CnCn* cyclic molecules are only present in even–even and odd–odd configurations. Conversely, *CnCm* asymmetric molecules consist of even–even + 2 or odd–odd + 2 integers of CH_2_ units (see Supplementary Tables S1–S6). As a continuation of this discussion, we will examine 51 double-branched cyclic molecules. To bind gold electrodes to these terminal groups, different binding energies are required. Direct carbon anchors have a binding energy of 1.9 eV, followed by thiol anchors at 1.7 eV, and amine anchors at 0.3 eV (see Supplementary Figure S1).

It is vital to describe the STM-BJ experimental setup and the statistical methods used to measure the conductance data to validate the DFT predictions presented here. This technique measures the conductance of a single molecule by periodically forming and rupturing the molecular junction via a ‘tapping’ process in which the tip contacts the substrate surface and then retracts. The junction electrodes utilized in this technique are typically composed of a gold substrate paired with a gold STM tip [22,33,48]. In this experiment, Gold is generally selected as the substrate because it is the most suitable electrode material owing to its malleability, high conductivity, and strong affinity for most functional groups. Moreover, it prevents the formation of an oxide layer on its surface when exposed to air by resisting surface passivation. Additionally, different chemical anchor groups could be used to ensure that potential molecules are presented within junctions. At electrode surfaces, molecules tend to aggregate, which results in the formation of metallic filaments during the development process [49]. When the filament breaks, molecules may become trapped in newly formed electrode gaps, creating molecular junctions. The current gradually decreases until it reaches noise levels when the junction breaks. As a result of the continuous retraction of the tip, the length of the junction eventually exceeds the geometric distance that the molecule can cover. It is estimated that a statistically significant dataset can be generated by repeating the aforementioned steps thousands of times. The collected results can be presented as a 1D histogram, from which the molecule’s conductance is obtained from the most prominent peak. Moreover, the collected data can be represented as a 2D heat map, in which the high-conductance cloud corresponds to single-molecule properties.

The conductance-distance and current traces could provide information about the chemical and physical processes occurring at the junction by revealing the length and structure of the conductance plateau [50]. These data facilitate the understanding of single-molecule chemistry, physics, and junction behavior. It is possible to classify and efficiently sort heterogeneous conductance measurements from any single molecule into several categories using machine learning algorithms. In addition to illustrating molecular-level details and validating results, computational models can be used to explore the electrical properties of molecular junctions, such as conductance. However, DFT methods are unable to accurately predict the electrode Fermi level and the orbital relative energies of molecules, which are critical for predicting molecular conductance. This issue may be addressed by the Quantum Circuit Rules, which are simple methods for quickly estimating single-molecular conductance and can be used as an empirical resource to clarify anomalous conductance trends [50].

## 3. Results and Discussion

This research systematically modeled the charge transport properties of 51 cyclic alkane molecular junctions incorporating three distinct anchor groups. The modeling was performed by integrating Density Functional Theory (DFT) with quantum transport theory, specifically the Landauer–Büttiker formalism. This combined approach enables the calculation of the transmission function *T*(*E*), which determines the probability of electrons passing through the molecular junction, thereby predicting its conductance properties. Our aimed to establish clear structure–property relationships by analyzing how different anchor groups and the specific cyclic structures of the alkanes influence the efficiency of electron transport. At the terminal of Au-NH2, the covalent bond distance is 2.8 Å, while the Au–S distance is 2.3 Å, and the Au–C distance is 2.3 Å for the terminal Au–C, as illustrated in Supplementary Figure S1 (details can be found in Section S2 of the Supplementary Materials).

**Figure 1:**
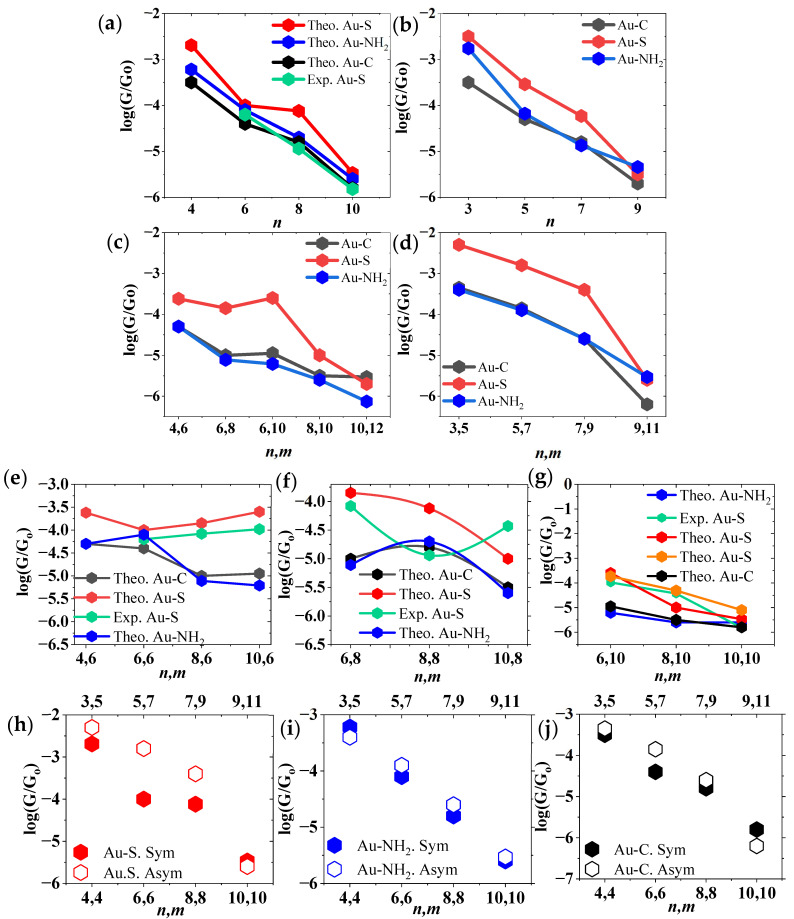
Length-dependent conductance of Au|cyclic molecule|Au single-molecule junctions with different molecular symmetries and anchoring groups. (**a**–**d**) Show the density functional theory (DFT)-calculated conductance as a function of molecular length for cyclic alkane molecules with different branch parity and symmetry: (**a**) symmetric cyclic molecules with even-numbered branches, (**b**) symmetric cyclic molecules with odd-numbered branches, (**c**) asymmetric cyclic molecules with even-numbered branches, and (**d**) asymmetric cyclic molecules with odd-numbered branches. In each case, conductance values are reported for three anchoring groups: thiol (Au–S, red), amine (Au–NH_2_, blue), and direct carbon contact (Au–C, black). Experimental scanning tunneling microscopy (STM) conductance data for thiol-terminated junctions are included, where available (green), for comparison [18]. (**e**–**g**) Comparison of theoretical and experimental logarithmic conductance values as a function of the total number of carbon atoms for cyclic molecules with fixed branch lengths: (**e**) 6-unit branches, (**f**) 8-unit branches, and (**g**) 10-unit branches. DFT results for thiol, amine, and direct carbon anchoring are shown as red, blue, and black hexagons, respectively. Experimental STM data for thiol-anchored junctions (green hexagons) and previously reported theoretical results (orange hexagons) are also included for comparison [18,19]. (**h**–**j**) Summary of logarithmic electrical conductance for symmetric and asymmetric cyclic alkane molecules featuring a constant upper carbon branch and a variable lower branch with three different anchor groups: (**h**) thiol (–SH), (**i**) amine (–NH_2_), and (**j**) direct carbon (–C) anchoring groups. Symmetric cyclic molecules (lower x-axis) and asymmetric cyclic molecules (upper x-axis) are distinguished by different hexagon colors. For all anchoring groups, symmetric and asymmetric molecules with identical carbon counts exhibit similar conductance values (see Supplementary Section S7 and Figures S17–S22 for detailed individual plots).

This theoretical DFT investigation examines charge transport in symmetric cyclic molecules within Au–Au junctions (please refer to Supplementary Section S4 and Figures S3–S14 for more details). The electrical conductance (*G*) is critically dependent on the terminal anchor group, with a distinct trend observed: Au–S thiol contacts exhibit the highest conductance, followed by Au–NH_2_ amine linkages, while direct Au–C covalent bonds show the lowest conductance. This trend is attributed to the strength and electronic coupling of the electrode–molecule interface, where the robust, resonant hybridization of the Au–S bond facilitates superior charge transport compared to the dative Au–NH_2_ bond and the less optimally hybridized Au–C bond. As shown in Figure 1a, symmetric cyclic molecules with an even number of CH_2_ units in each branch exhibit an apparent decrease in conductance as molecular size increases (*n* = 4, 6, 8, and 10). Experimental STM-BJ results for Au–S–anchored systems (*n* = 6, 8, and 10) are included for comparison. Although the experimental intercept deviates slightly from the theoretical prediction, the measured decay trend (slope) agrees well with the DFT-calculated behavior. Notably, the conductance differences between systems become less pronounced as the ring diameter increases; in particular, all anchoring groups yield nearly identical conductance values for *C*_10_*C*_10_. This convergence is consistently observed in both theoretical and experimental data, suggesting that, at sufficiently large ring sizes, the influence of the anchoring group on σ-mediated transport becomes minimal.

Figure 1b presents a complementary case, showing the conductance behavior of symmetric cyclic molecules with both branches containing an identical odd number of CH_2_ units (*n* = *m*), specifically *C*_3_*C*_3_, *C*_5_*C*_5_, *C*_7_*C*_7_, and *C*_9_*C*_9_. Similar to the even-numbered systems, these odd-numbered rings display the expected trend of decreasing conductance with increasing molecular cavity size. Importantly, for the largest structure in this series (*C*_9_*C*_9_), the calculated conductance converges to nearly the same value regardless of the anchoring group employed. This convergence further reinforces the observation that, for sufficiently large saturated cyclic systems, the influence of anchor chemistry on σ-mediated transport becomes increasingly negligible.

For asymmetric cyclic molecules—where the upper and lower branches differ in the number of CH_2_ units (*n* ≠ *m*)—we adopt a simplified notation for clarity. Even-numbered systems are labeled as $C_{2n}C_{m=2n+2}$ (e.g., *C*_4_*C*_6_, *C*_6_*C*_8_, and *C*_6_*C*_10_, *C*_8_*C*_10_, *C*_10_*C*_12_), whereas odd-numbered systems are denoted as $C_{2n-1}C_{m=2n+1}$ (e.g., *C*_3_*C*_5_, *C*_5_*C*_7_, *C*_7_*C*_9_, and *C*_9_*C*_11_). As illustrated in Figure 1c for even-numbered asymmetric molecules—and Figure 1d presents odd-numbered asymmetric molecules—the conductance (*G*) exhibits a classical length dependence, decreasing systematically with increasing molecular size. Furthermore, the anchoring groups exert a pronounced effect on the absolute conductance values. Junctions formed with thiol anchors (Au–S) consistently yield the highest conductance, while amine (Au–NH_2_) and direct carbon contacts (Au–C) produce lower and nearly comparable conductance values.

Regarding the relationship between molecular dimensions and conductance, amine anchors form a well-defined, localized binding site, leading to a relatively predictable exponential decay in conductance as molecular length increases. However, their conductance values are typically lower due to the higher tunneling barrier associated with the nitrogen–gold interface. In contrast, direct carbon anchoring affords stronger electronic coupling to the electrode, yielding higher conductance and a shallower length-dependent decay. Additionally, the conductance of carbon-anchored systems is more sensitive to molecular diameter and orientation, as larger cross-sectional areas enhance orbital overlap and improve charge-injection efficiency. Thus, while amine anchors offer synthetic convenience and selectivity, direct carbon contacts provide superior charge-transport characteristics by maximizing both electronic coupling and conformational stability.

This DFT investigation examined the electrical conductance of cyclic molecules featuring a constant upper carbon branch and a variable lower branch, using thiol, amine, and direct carbon anchors. The systems were categorized into three units based on the common branch length. The 6-unit branch (common *C*_6_ branch), which includes molecules such as *C*_4_*C*_6_, *C*_6_*C*_6_, *C*_8_*C*_6_, and *C*_10_*C*_6_, revealed an anchor-dependent dichotomy: thiol anchors exhibited a non-conventional trend where conductance increased with cavity size, while amine and direct carbon anchors followed the conventional pattern of decreasing conductance with increasing molecular length, as shown in Figure 1e. The 8-unit branch (common *C*_8_ branch), comprising structures such as *C*_6_*C*_8_, *C*_8_*C*_8_*,* and *C*_10_*C*_8_, showed a downward parabolic trend for amine and direct carbon anchors, with maximum conductance at the symmetric *C*_8_*C*_8_ ring, a finding corroborated by experimental data, as shown in Figure 1f. Finally, the 10-unit branch (common *C*_10_ branch), which includes molecules such as *C*_6_*C*_10_, *C*_8_*C*_10_, and *C*_10_*C*_10_, primarily adhered to the conventional trend of decreasing conductance with increasing size. Notably, the conductance of the largest symmetric ring (*C*_10_*C*_10_) was invariant to the anchor group, as illustrated in Figure 1g. These DFT predictions are consistently supported by independent experimental and theoretical studies, validating the computational approach.

Although our DFT calculations reproduce the experimental conductance trends very well, some quantitative differences between theory and experiment remain. Such discrepancies are common in single-molecule transport studies and arise from physical effects that are not fully captured by standard DFT-based approaches. In experiments, conductance can be influenced by environmental factors such as solvent interactions, thermal fluctuations, and molecular conformational dynamics, which are not explicitly included in the theoretical model. In addition, atomic-scale variations in electrode geometry and molecule–electrode contacts occur naturally from junction to junction in STM break-junction measurements. Limitations in Fermi-level alignment within conventional DFT methods may also contribute to systematic shifts in the calculated conductance. Nevertheless, the close agreement in conductance trends and relative ordering across the molecular series demonstrates that the theoretical framework reliably captures the essential structure–transport relationships governing charge transport in molecular junctions.

To better understand the conductance patterns of cyclic molecules, a comparison was made between the electrical conductance of symmetric and asymmetric molecules possessing an identical total number of carbon atoms. In this analysis, even-numbered symmetric rings ($C_{n}C_{n}$) were compared to odd-numbered asymmetric rings ($C_{2n-1}C_{m=2n+1}$), such as *C*_8_*C*_8_ versus *C*_7_*C*_9_ and *C*_6_*C*_6_ versus *C*_5_*C*_7_. Although the overall conductance (*G*) values are approximately similar for molecules with identical carbon counts, a closer inspection reveals that the asymmetric molecules consistently exhibit higher conductance, as illustrated in Figure 1h–j. This phenomenon can be rationalized by the fact that the relaxed geometries of asymmetric molecules possess smaller effective diameters than their symmetric counterparts of equal atomic composition. For instance, the asymmetric *C*_7_*C*_9_ molecule with a thiol anchor has a diameter of 10.3 Å, which is less than the 10.45 Å diameter of the symmetric *C*_8_*C*_8_ molecule. Similarly, the diameter of the asymmetric *C*_5_*C*_7_ molecule (7.4 Å) is smaller than that of the symmetric *C*_6_*C*_6_ molecule (8.05 Å). These observations reinforce the correlation between reduced molecular diameter and enhanced conductance (see Supplementary Section S5 and Figure S15 for additional details).

The dominance of molecular diameter over anchoring-group chemistry in determining conductance reflects a transition from single-path tunneling to multi-channel transport. A larger molecular diameter provides a greater number of parallel σ-conduction pathways, effectively increasing the number of channels available for charge transport. However, the final conductance is not determined by channel count alone: quantum interference (QI) between these pathways governs the net transmission. Constructive QI enhances conductance by reinforcing electron amplitudes across channels, whereas destructive QI suppresses conductance by canceling them. Thus, while the molecular diameter dictates the channel capacity, QI ultimately defines how these channels interact coherently to produce the measured conductance. Collectively, these observations demonstrate that σ-transport in cyclic frameworks is determined by both geometric constraints and quantum-coherent effects, rather than solely by anchoring chemistry. The Supporting Information (Section S6, Figure S16) presents a consolidated comparison of these structure–conductance relationships, providing an integrated visualization that reinforces and contextualizes the mechanistic interpretations discussed in this section.

## 4. Conclusions

In conclusion, this comprehensive theoretical DFT investigation examined the single-molecule conductance of 51 organic cyclic molecules, probed with three distinct terminal anchor groups. Counterintuitively, different trends can be observed across different molecular series: the 6-unit branch exhibits a non-conventional deviation, the 8-unit branch follows a parabolic relationship, and only the large-diameter 10-unit branch adheres to the conventional monotonic decay. Furthermore, a key finding is that symmetric and asymmetric cyclic molecules with identical total counts of CH_2_ units exhibit remarkably similar conductance values across all three anchor types. This indicates that, for these systems, the global molecular structure—specifically, the total molecular size—exerts a more dominant influence on electron transport than the local structural symmetry, providing a crucial design principle for future molecular electronic components.

## Figures and Tables

**Table 1:** Examples of thiol-terminated cyclic molecular systems, categorized by symmetry and backbone parity. The upper table displays symmetric molecules (odd–odd and even–even), while the lower table shows asymmetric molecules (odd–odd + 2 and even–even + 2). For clarity, only eight representative structures are shown here. Comprehensive data on all studied molecules can be found in Supplementary Tables S1–S6.

Symmetric Cyclic Thiol Anchor			
*n,n*	Molecule	*n,n*	Molecule
3,3	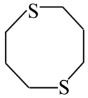	4,4	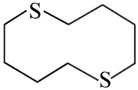
7,7	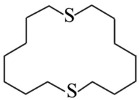	8,8	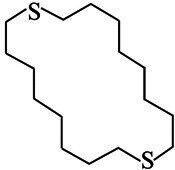
**Asymmetric Cyclic Thiol Anchor**			
***n,m***	**Molecule**	***n,m***	**Molecule**
3,5	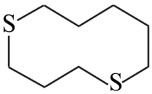	4,6	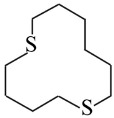
9,11	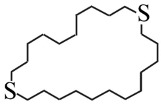	8,10	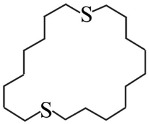

## Data Availability

The original contributions presented in this study are included in the article/Supplementary Material. Further inquiries can be directed to the corresponding authors.

## Supplementary Materials

The following supporting information can be downloaded at https://www.mdpi.com/article/10.3390/nano16020083/s1. Refs. [51,52,53,54,55,56,57,58,59,60,61,62,63,64,65,66,67,68,69,70,71,72,73,74,75,76] are cited in the Supplementary materials.

## Abbreviations

DFT	Density Functional Theory
STM-BJ	Scanning Tunneling Microscope–Break Junction
MCBJs	Mechanically Controllable Break Junctions
QI	Quantum Interference
NEGF	Non-equilibrium Green’s function
