# Supplementary material for: Tuning Nanoscale Conductance in Cyclic Molecules via Molecular Length and Anchoring Groups
Source: Nanomaterials (Basel). 2026 Jan 7;16(2):83. doi: 10.3390/nano16020083 (PMC12844402; doi:10.3390/nano16020083)
Supplement: Supplementary file 1 [file nanomaterials-16-00083-s001.zip › nanomaterials-4077816-supplementary.pdf]

S1. Geometry of isolated cyclic alkane molecules

As According to Tables S1-S6, DFT code (SIESTA)[1-6] was utilized to obtain fully relaxed geometry for isolated cyclic molecules. As shown in Tables S1-S2, 17 symmetric and asymmetric cyclic molecules terminated with direct carbon are presented in their fully relaxed and isolated conformations, respectively. (Odd-odd) rings are examples of symmetric molecules. From (C3C3), two CH2 units were added to each branch until n = 9 (C9C9) was reached. In addition, (even-even) rings follow the same rule. In Table S1, we begin with (C4C4) and continue until (C10C10). Asymmetric cyclics, as shown in Table S2, are constructed by starting with (C3C5) and adding two CH2 units for each branch until $n=9$, $m=11$ (C9C11). In the same way, (even-even+2) molecules begin with (C4C6) and end with (C10C12).

Similarly, in Tables S3-S4, symmetric and asymmetric rings are now terminated with thiol anchors. As can be seen in Tables S5-S6, cyclic molecules are now terminated with amine anchors.

Table S1. Fully relaxed symmetric cyclic rings containing direct carbon. The (odd-odd) and (even-even) molecules represent symmetric cyclic molecules. The (odd-odd) and (even-even) molecules represent symmetric cyclic molecules.

**Symmetric-Direct C anchor**			
**Molecule**	**Odd-odd**	**Molecule**	**Even-even**
Cyclooctane (ref.7) (C3C3)	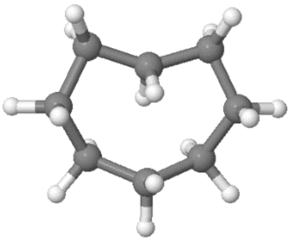	Cyclodecane (ref.10) (C4C4)	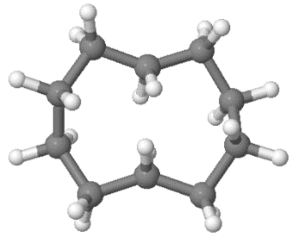
Cyclododecane (ref.8) (C5C5)	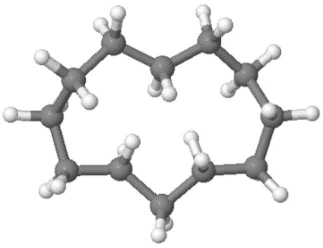	Cyclotetradecane (ref.11) (C6C6)	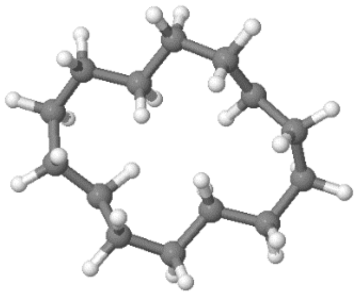
Cyclohexadeane (ref.9) (C7C7)	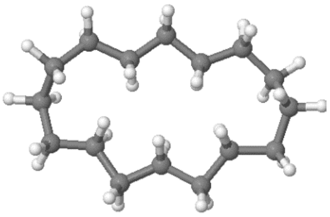	Cyclooctadecane (ref.9) (C8C8)	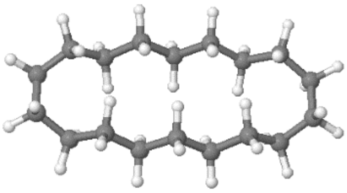
Cycloeicosane (ref.9) (C9C9)	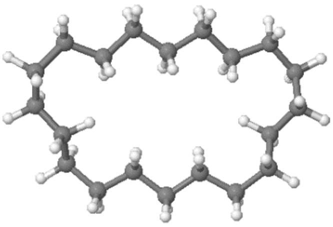	Cyclodocosane (ref.12) (C10C10)	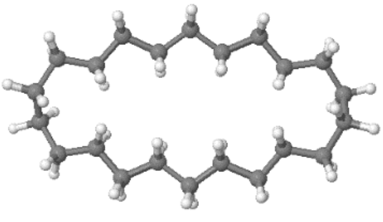

Table S2. Fully relaxed asymmetric cyclic rings containing direct carbon. The (odd-odd+2) and (even-even+2) molecules refer to asymmetric cyclic molecules.

**Asymmetric-Direct C anchor**			
**Molecule**	**Odd-odd+2**	**Molecule**	**Even-even+2**
Cyclooctane (ref.10) (C3C5)	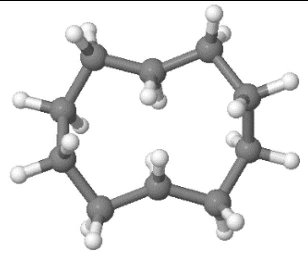	Cyclododecane (ref.8) (C4C6)	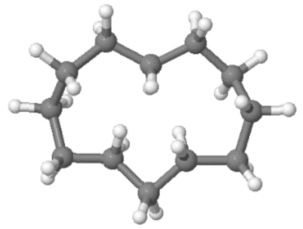
Cyclotetradecane (ref.11) (C5C7)	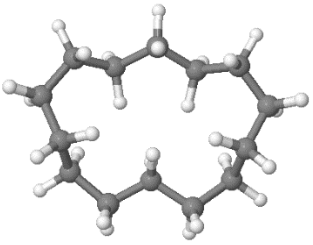	Cyclohexadecane (ref.9) (C6C8)	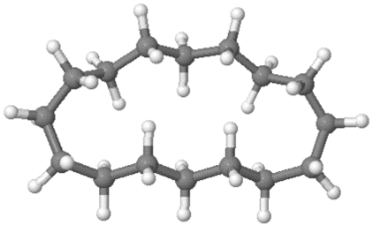
Cyclooctadecane (ref.9) (C7C9)	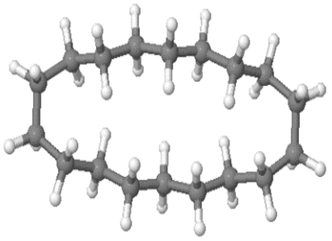	Cyclooctadecane (ref.9) (C6C10)	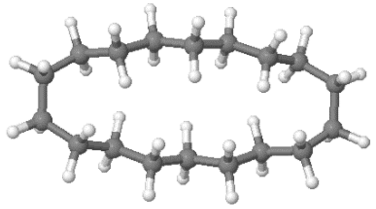
Cyclodocosane (ref.12) (C9C11)	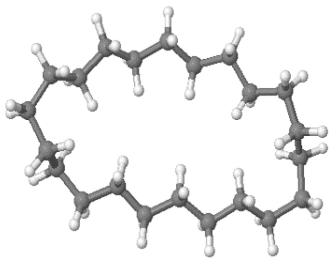	Cycloeicosane (ref.9) (C8C10)	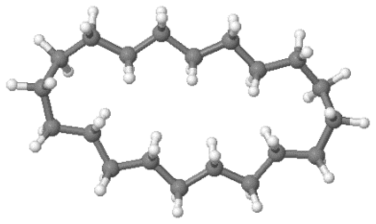
		Cyclotetracosane (ref.13) (C10C12)	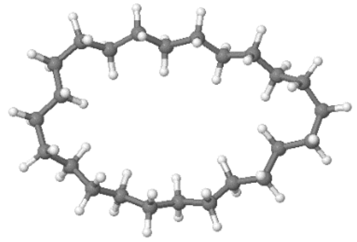

Table S3. Fully relaxed symmetric cyclic rings containing thiol anchor. The (odd-odd) and (even-even) molecules represent symmetric cyclic molecules.

**Symmetric-Thiol anchor**			
**Molecule**	**Odd-odd**	**Molecule**	**Even-even**
1,5-Dithiocane<br>(ref.14)<br>(C3C3)	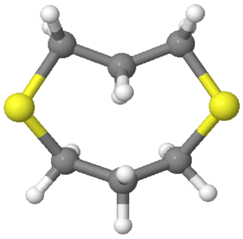	1,6-dithiecane<br>(ref.17)<br>(C4C4)	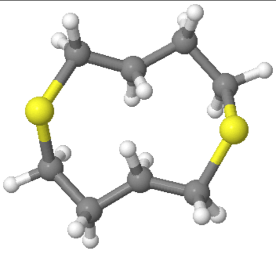
1,7-Dithiacyclododecane<br>(ref.15)<br>(C5C5)	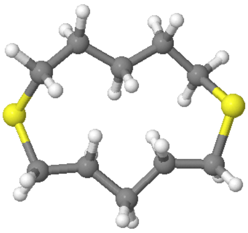	1,8-Dithia-cyclotetradecan<br>(ref.18-19)<br>(C6C6)	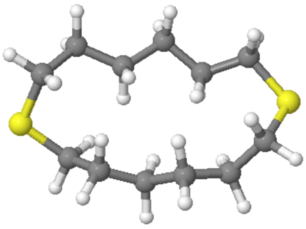
1,9-Dithia-cyclohexadecan<br>(ref.16)<br>(C7C7)	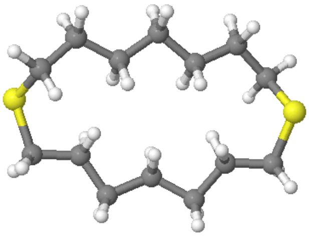	1,10-Dithia-cyclooctadecan<br>(ref.19)<br>(C8C8)	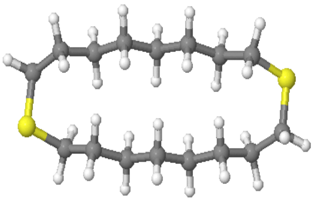
(C9C9)	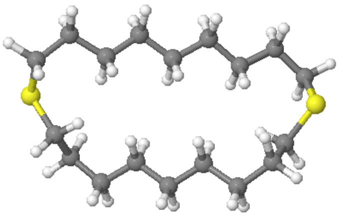	(C10C10)<br>(ref.19)	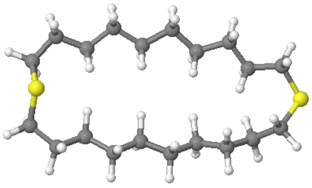

Table S4. Fully relaxed asymmetric cyclic rings containing thiol anchor. The (odd-odd+2) and (even-even+2) rings refer to asymmetric cyclic molecules.

Asymmetric-Thiol anchor			
**Molecule**	**Odd-odd+2**	**Molecule**	**Even-even+2**
(C3C5)	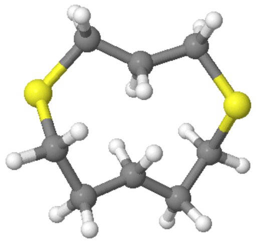	1,6-Dithiacyclododecane (ref.20) (C4C6)	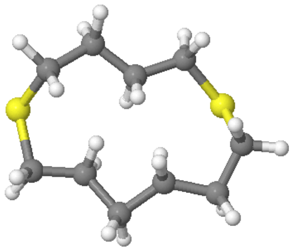
(C5C7)	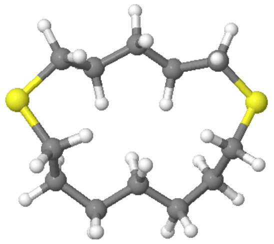	(C6C8) (ref.19)	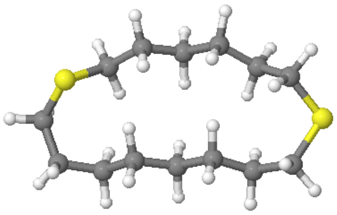
(C7C9)	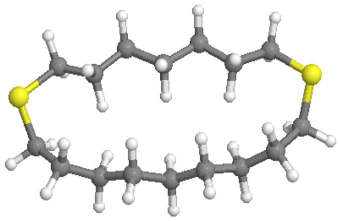	1,8-Dithiacyclooctadecane (ref.19) (C6C10)	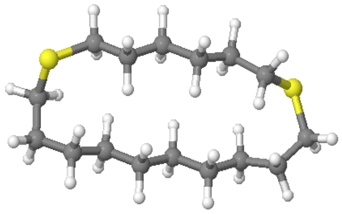
(C9C11)	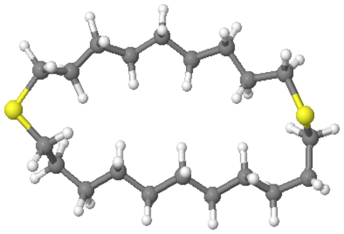	(C8C10) (ref.19)	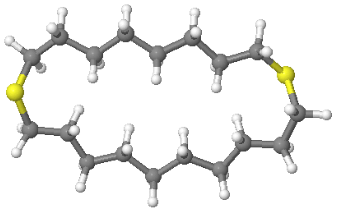
		(C10C12)	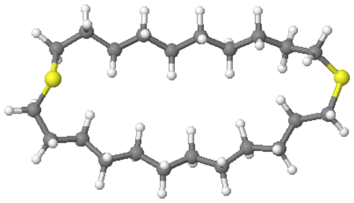

Table S5. Fully relaxed symmetric cyclic rings containing amine anchor. The (odd-odd) and (even-even) rings represent symmetric cyclic molecules.

Symmetric-Amine anchor			
**Molecule**	**Odd-odd**	**Molecule**	**Even-even**
1,5-Diazacyclooctane<br>(ref.21)<br>(C3C3)	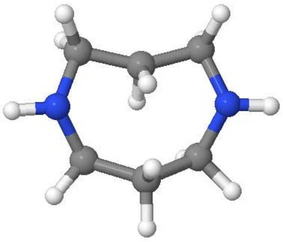	1,6-Diazecane<br>(ref.25)<br>(C4C4)	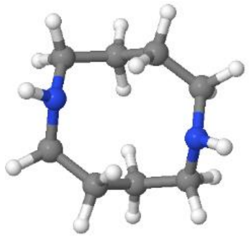
1,7-Diazacyclododecane<br>(ref.22)	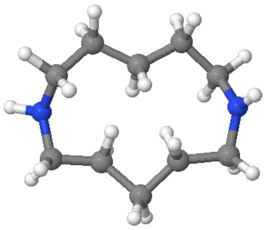	1,8-Diazacyclotetradecan (C6C6)<br>(ref.26)	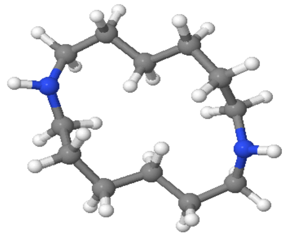
1,9-Diazacyclohexadecane<br>(ref.23)<br>(C7C7)	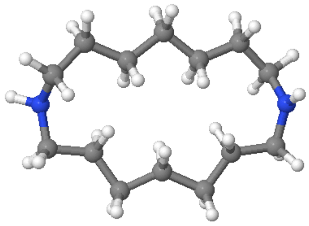	1,10-Diazacyclooctadecane<br>(ref.27)<br>(C8C8)	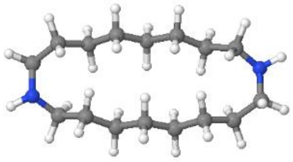
1,11-Diaza-cycloeikosan<br>(ref.24)<br>(C9C9)	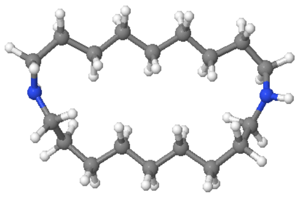	1,12-Diazacyclodocosane<br>(ref.28)<br>(C10C10)	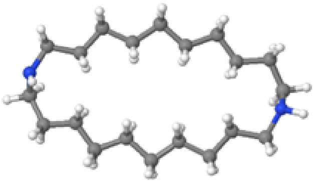

Table S6. Fully relaxed asymmetric cyclic rings containing amine anchor. The (odd-odd+2) and (even-even+2) rings refer to asymmetric cyclic molecules.

Asymmetric-Amine anchor			
**Molecule**	**Odd-odd+2**	**Molecule**	**Even-even+2**
1,5-Diazecane<br>(ref.29)<br>(C3C5)	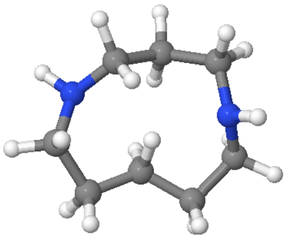	1,6-Diazacyclododecane<br>(ref.31)<br>(C4C6)	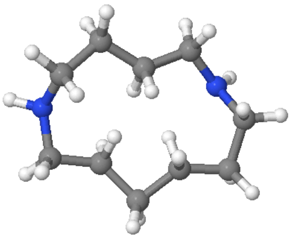
1,7-Diazacyclotetradecane<br>(ref.29)<br>(C5C7)	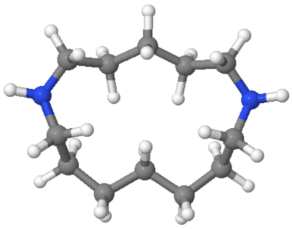	(C6C8)	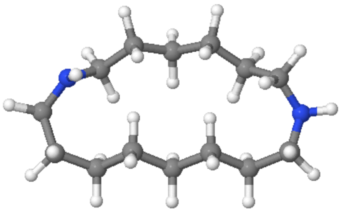
(C7C9)	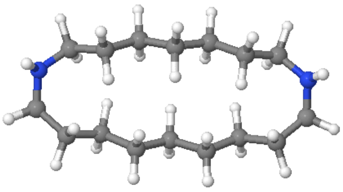	1,8-Diazacyclooctadecane<br>(ref.32)<br>(C6C10)	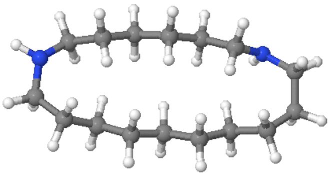
(C9C11)	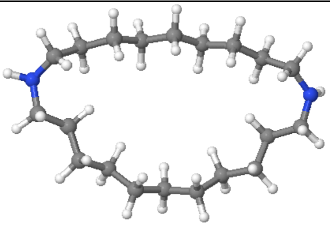	(C8C10)	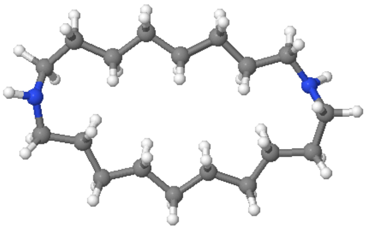
		(C10C12)	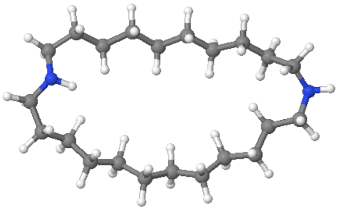

S2. Binding energy of four terminal groups on gold

As part of the calculations to determine the optimal distance between the cyclic molecules bound to the gold surfaces with three different terminal groups (Au-NH2, Au-S, and Au-C), we used DFT and counterpoise methods to eliminate basis set superposition errors (BSSE). A binding distance can be calculated by measuring the distance between the gold surface and the molecule's terminated end group or atom. SIESTA was used to determine the ground-state energy of the overall system, denoted by E_{AB}^{AB}. Gold leads are composed of six layers, each containing 30 atoms. In SIESTA, ghost atoms are used to calculate on a fixed basis. Using a fixed basis, each molecule's the binding energy can be calculated as follows: [1,5]

$$\text{Binding Energy} = E_{AB}^{AB} - E_{A}^{AB} - E_{B}^{AB} \qquad \text{(S1)}$$

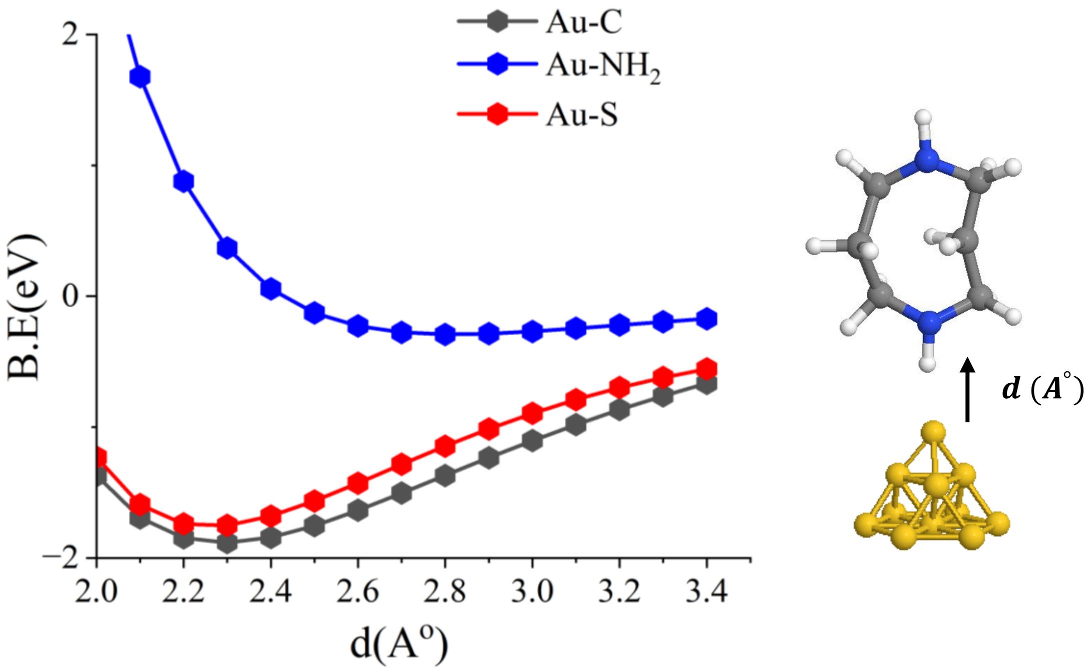

Figure S1. Binding energy of cyclic molecules to gold as a function of molecule-contact distance. The equilibrium distance (i.e. the minimum of the binding energy curve) is found to be approximately 2.3, 2.3 and 2.8 Å for Au-C, Au-S,and Au- NH_2 (top to bottom). Key: C = grey, H = white, N = blue, Au = dark yellow.

S3. Optimised DFT structures of compounds in their Junctions

These are some examples of optimised DFT structures of cyclic rings at their junctions, as illustrated in Figure S2.

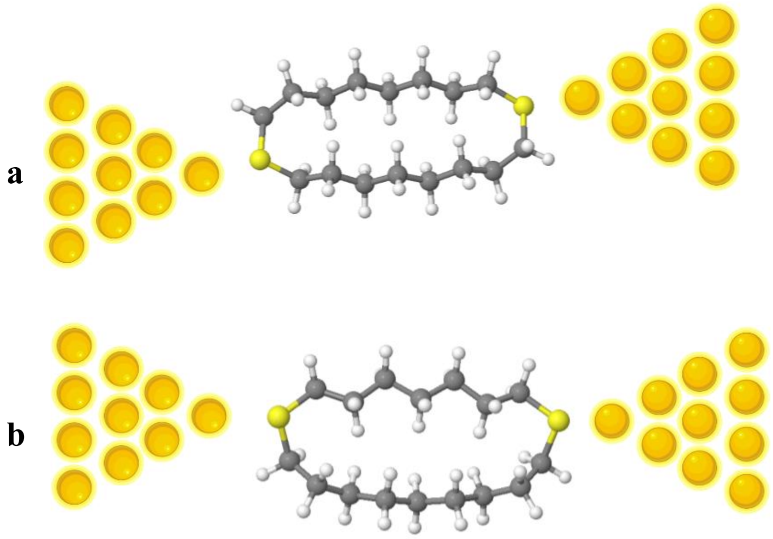

Figure S2. Examples of cyclic molecules in Au|cyclic molecules|Au junctions: **(a)** is a double-branch n,m = 8,8 cyclic molecule with (Au-S), while (**b**) is a double-branch n,m = 7,9 cyclic molecule with the teminal anchor group.

S4. Conductance comparison between cyclic molecules of different terminal groups

The purpose of this section is to compare the conductance G of cyclic molecules containing three different terminal groups: direct carbon Au-C, thiols Au-S, and amines Au-NH2. There are three sets of transmission coefficient curves for cyclic molecules with Au-C shown in Figures S3 through S6; however, Figures S7 through S10 exhibit the same curves with Au-S. Figures S11 through S14 present transmission coefficient curves for cyclic molecules with Au-NH2.

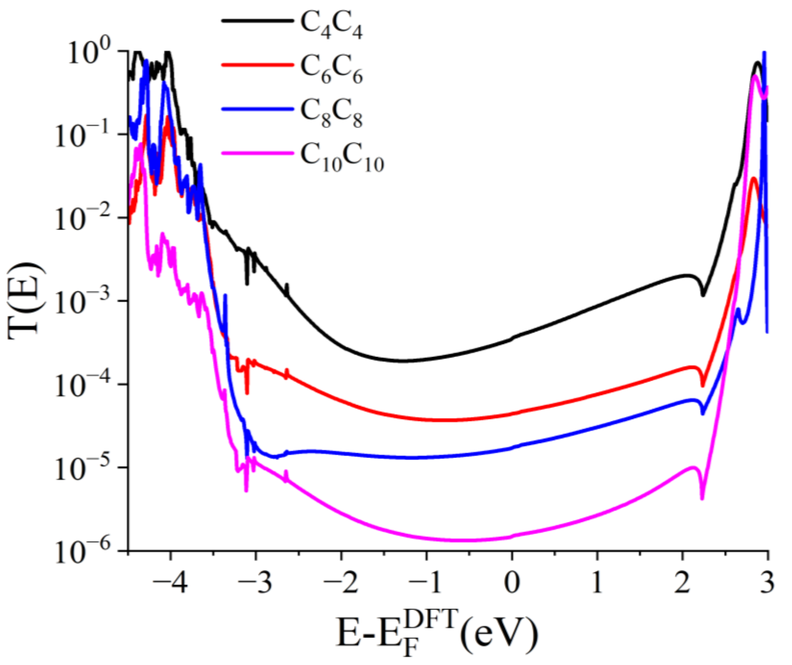

Figure S3. Transmission coefficient curves of cyclic molecules with (Au-C)**.** Even-even cyclic molecules transmission coefficients T(E) against electron energy E for C_4C_4 (black line), C_6C_6 (red line), C_8C_8 (blue line) and $C_{10}C_{10}$ (pink line).

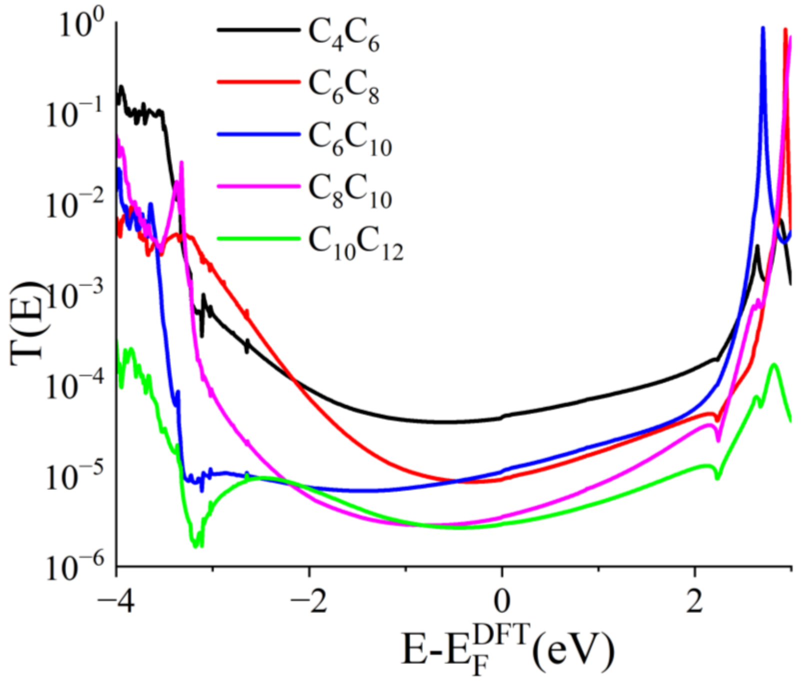

Figure S4. Transmission coefficient curves of cyclic molecules molecules with (Au-C)**.** Even-even+2 cyclic molecules molecules transmission coefficients T(E) against electron energy E for C_4C_6 (black line), C_6C_8 (red line), C_6C_{10} (blue line), C_8C_{10} (pink line) and $C_{10}C_{12}$ (green line).

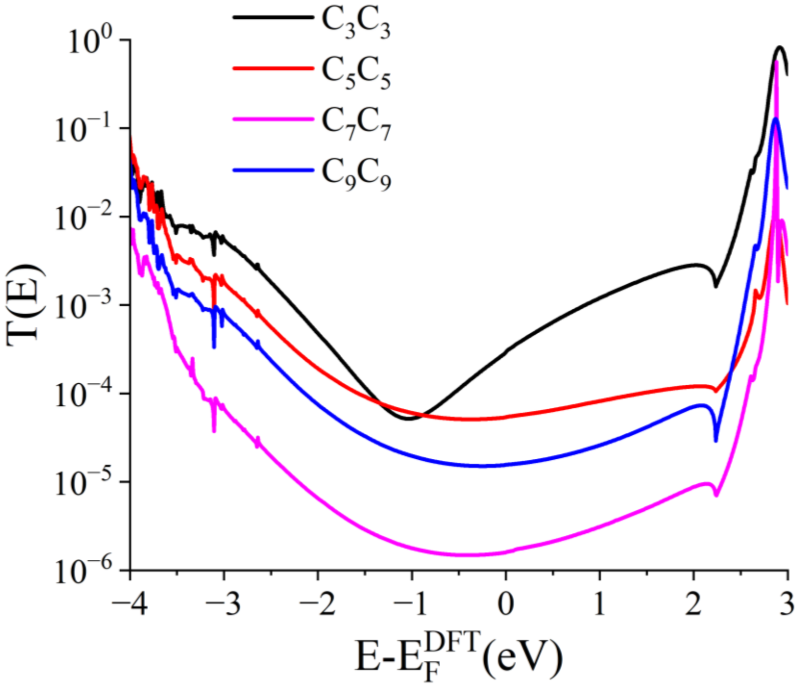

Figure S5. Transmission coefficient curves of cyclic molecules with (Au-C)**.** Odd-odd cyclic molecules transmission coefficients T(E) against electron energy E for C_3C_3 (black line), C_5C_5 (red line), C_7C_7 (pink line) and C_9C_9 (blue line).

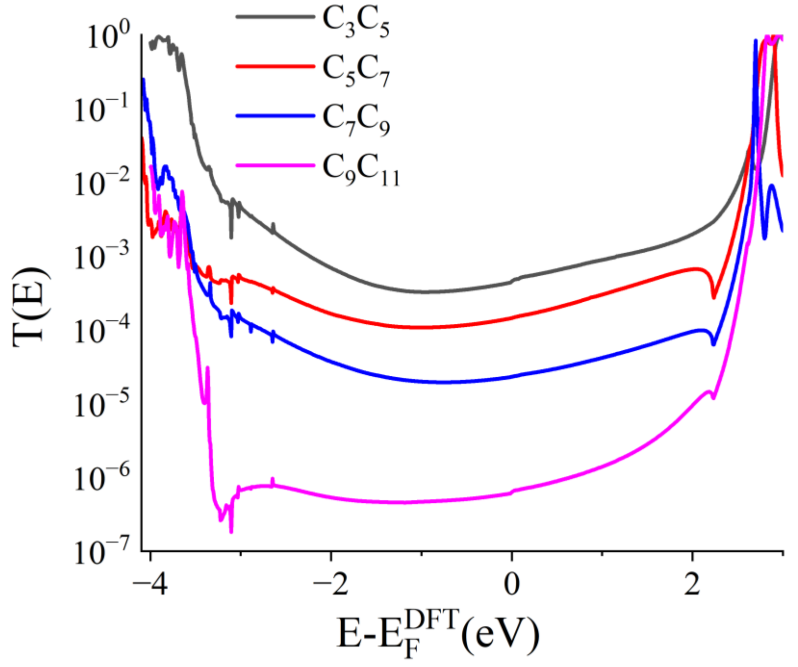

Figure S6. Transmission coefficient curves of cyclic molecules with (Au-C)**.** Odd-odd+2 cyclic molecules transmission coefficients T(E) against electron energy E for C_3C_5 (black line), C_5C_7 (red line), C_7C_9 (blue line) and C_9C_{11} (pink line).

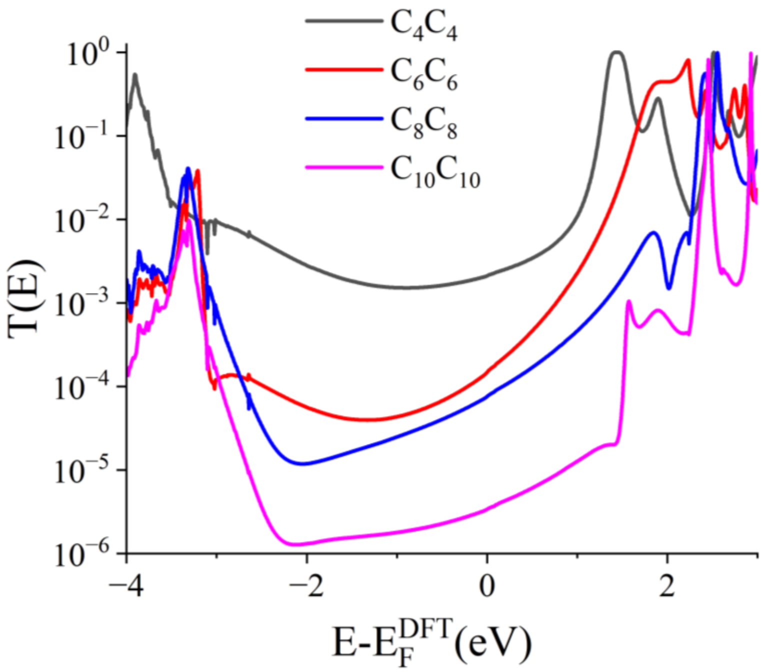

Figure S7. Transmission coefficient curves of cyclic moleculeswith (Au-S)**.** Even-even cyclic molecules transmission coefficients T(E) against electron energy E for C_4C_4 (black line), C_6C_6 (red line), C_8C_8 (blue line) and $C_{10}C_{10}$ (pink line).

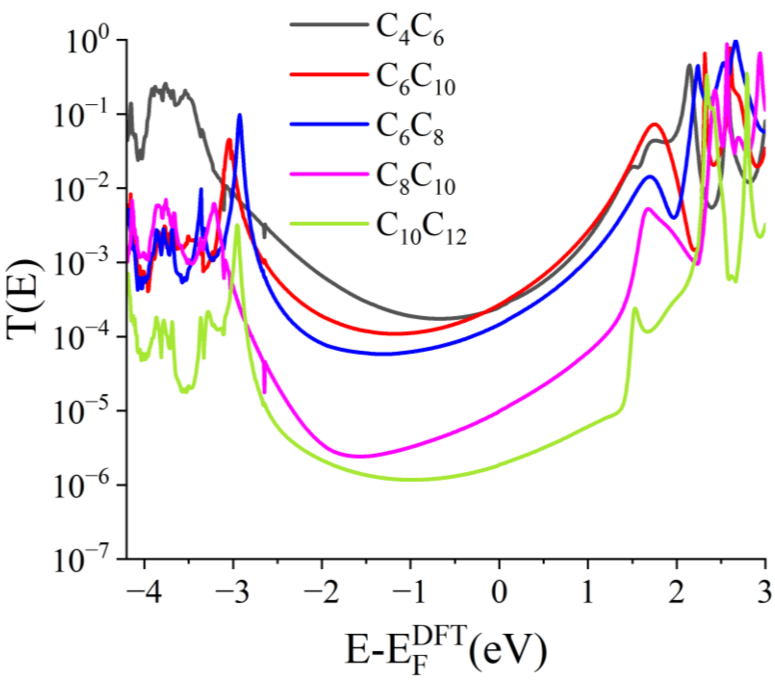

Figure S8. Transmission coefficient curves of cyclic molecules with (Au-S)**.** Even-even+2 cyclic molecules transmission coefficients T(E) against electron energy E for C_4C_6 (black line), C_6C_{10} (red line), C_6C_8 (blue line), C_8C_{10} (pink line) and $C_{10}C_{12}$ (green line).

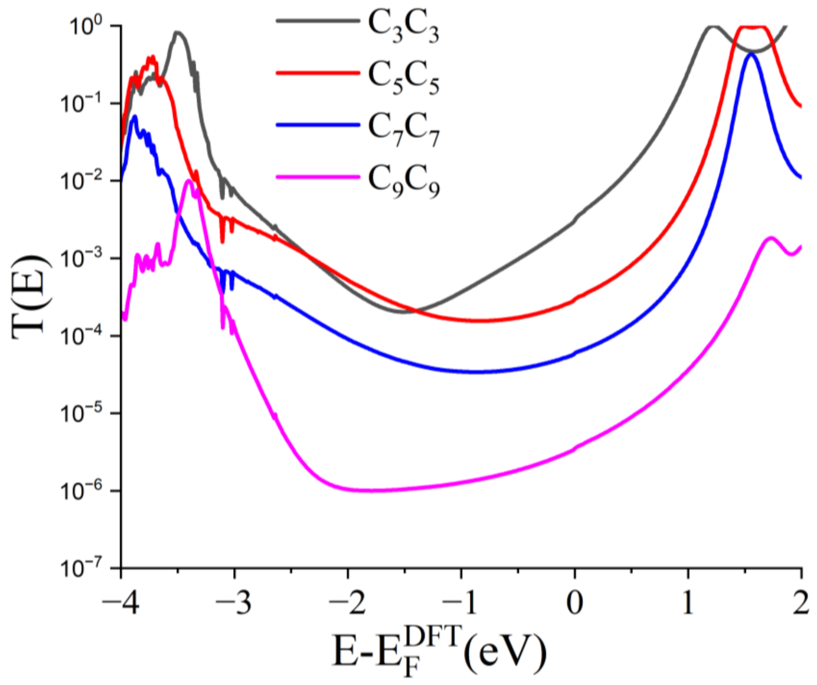

Figure S9. Transmission coefficient curves of cyclic molecules with (Au-S)**.** Odd-odd cyclic molecules transmission coefficients T(E) against electron energy E for C_3C_3 (black line), C_5C_5 (red line), C_7C_7 (blue line) and C_9C_9 (pink line).

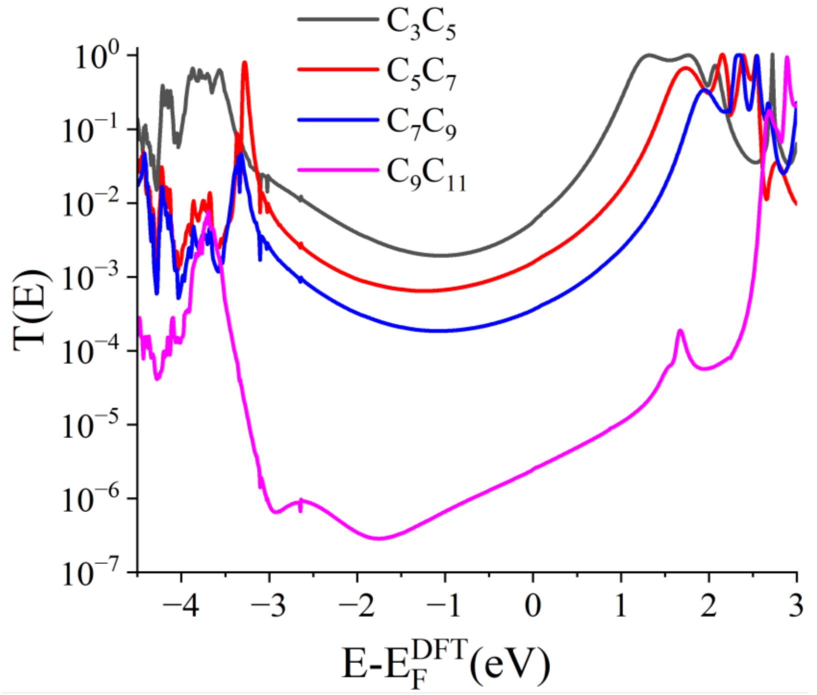

Figure S10. Transmission coefficient curves of cyclic molecules with (Au-S)**.** Odd-odd+2 cyclic molecules transmission coefficients T(E) against electron energy E for C_3C_5 (black line), C_5C_7 (red line), C_7C_9 (blue line) and C_9C_{11} (pink line).

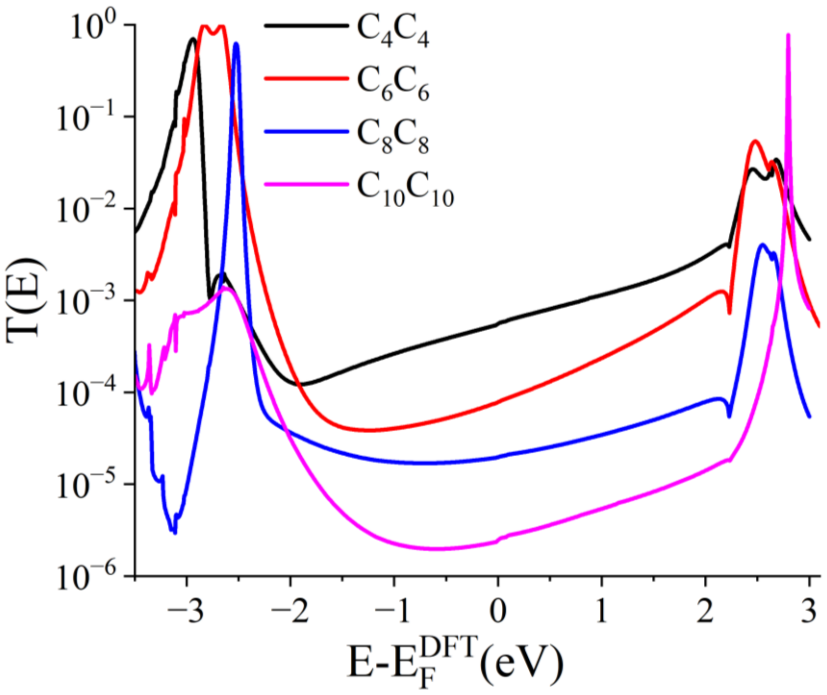

Figure S11. Transmission coefficient curves of cyclic molecules with (Au-NH_2). Even-even cyclic molecules transmission coefficients T(E) against electron energy E for C_4C_4 (black line), C_6C_6 (red line), C_8C_8 (blue line) and $C_{10}C_{10}$ (pinkline).

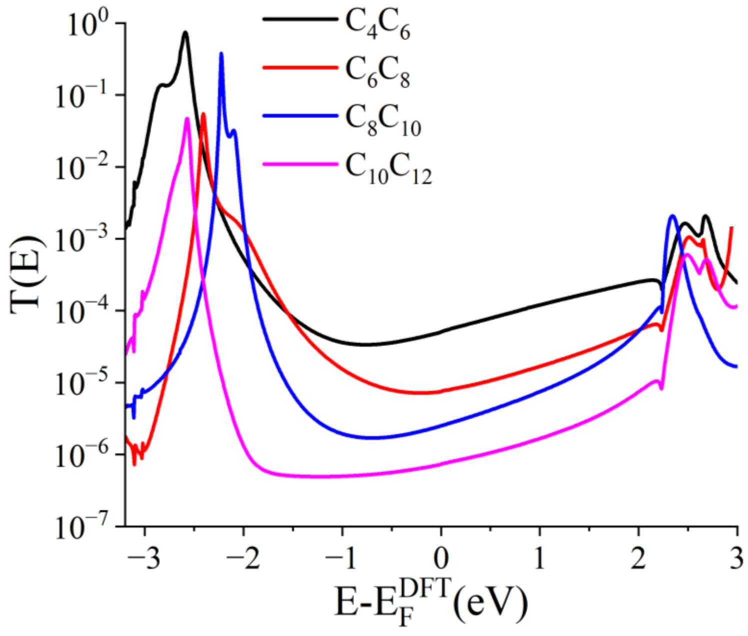

Figure S12. Transmission coefficient curves of cyclic molecules with (Au-NH_2)**.** Even-even+2 cyclic molecules transmission coefficients T(E) against electron energy E for C_4C_6 (black line), C_6C_{10} (red line), C_6C_8 (blue line), C_8C_{10} (pink line) and $C_{10}C_{12}$ (green line).

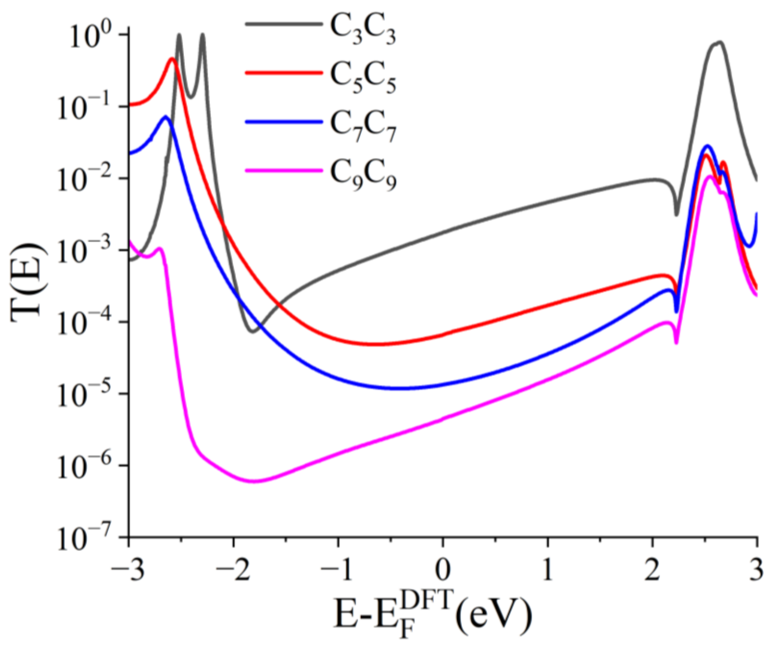

Figure S13. Transmission coefficient curves of cyclic molecules with (Au-NH_2)**.** Odd-odd cyclic molecules transmission coefficients T(E) against electron energy E for C_3C_3 (black line), C_5C_5 (red line), C_7C_7 (blue line) and C_9C_9 (pink line).

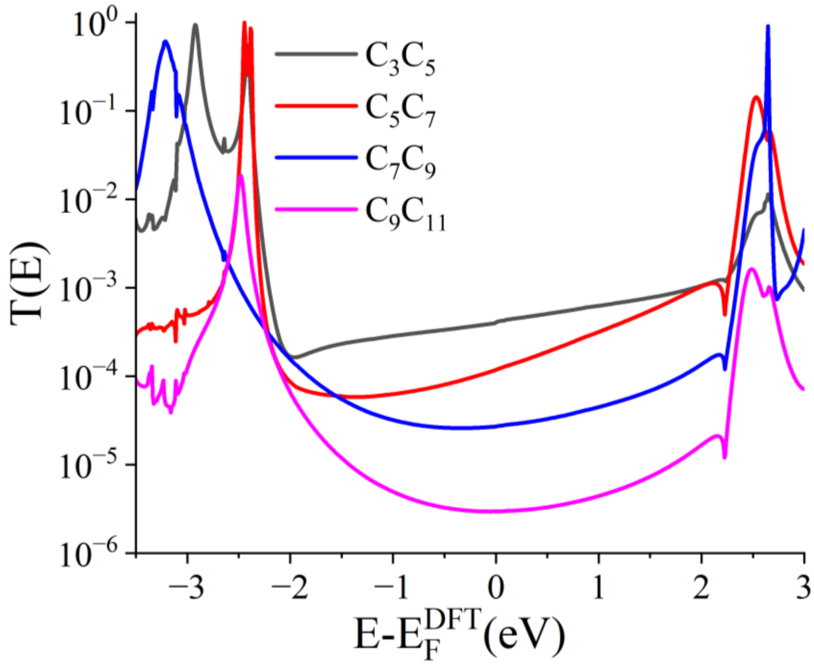

Figure S14. Transmission coefficient curves of cyclic molecules with (Au-NH_2)**.** Odd-odd+2 cyclic molecules transmission coefficients T(E) against electron energy E for C_3C_5 (black line), C_5C_7 (red line), C_7C_9 (blue line) and C_9C_{11} (pink line).

S5. The effect of diameter and length on molecular conductance

In this section, we demonstrate that conductance is affected by the diameter and length of relaxed organic cyclic molecules at their junctions. Two examples of asymmetric ring molecules with higher conductance than symmetric molecules, even when both contain the same number of methylene –CH_2 units, are shown in Figure S15.

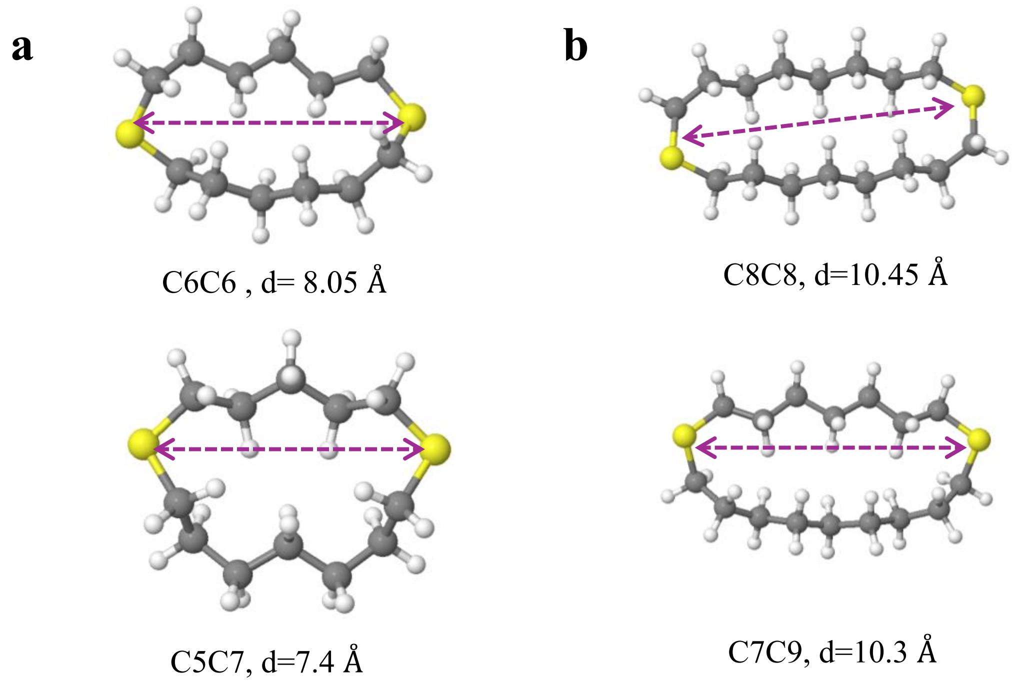

Figure S15. Examples of symmetric and asymmetric organic cyclic molecules with different diameters, with a thiol anchor group. Columns (**a**) and (**b**) illustrate symmetric and asymmetric molecules with varying diameter lengths, respectively. Note: The dashed purple arrow represents the diameter of the molecule.

S6. Summary Comparison of Conductance Trends

To complement the detailed conductance plots presented in the main manuscript (Figures 1–6), Figure S5provides a merged summary of all theoretical and experimental conductance values for the complete molecular dataset. This combined figure assists readers in visualizing the overarching structure–property relationships across the different molecular families.

As shown in Figure S16:

- Figure S16 (a-d) demonstrates that the conductance of both symmetric and asymmetric cyclic structures decreases systematically with increasing

molecular length, independent of the anchoring group employed—consistent with the conventional σ-tunneling trend.

- 6-unit branch molecules show thiol-dependent non-classical conductance behavior, with increasing conductance for larger rings—supported experimentally in Figure S16 (panel e).
- 8-unit branch molecules display a characteristic parabolic trend. Theoretical results exhibit a downward parabolic dependence, whereas the experimental data show an upward parabolic patteren as shown in Figure S16 (panel f). Importantly, the symmetric C8C8 molecule has nearly identical conductance values in both theory and experiment, underscoring the accuracy and reliability of our DFT calculations.
- 10-unit branch molecules show monotonic length-dependent decay typical of conventional σ-tunneling as shown in Figure S16 (panel g).
- Importantly, thiol-anchored junctions consistently exhibit higher conductance than amine-anchored systems, a behavior that aligns with widely established experimental and theoretical findings [33-36] demonstrating the stronger Au–S electronic coupling and more efficient charge injection compared with Au–NH_2 .
- Symmetric vs. asymmetric C_nC_m molecules fall within similar conductance envelopes regardless of the anchoring group, confirming that molecular diameter—rather than symmetry—plays the dominant role in determining σ-mediated charge transport in these systems, as shown in Figure S16 (panels h-j).

This consolidated visualization reinforces and clarifies the interpretations discussed in Section 3 of the main manuscript and provides a convenient reference for readers comparing theoretical predictions with experimental STM-BJ measurements.

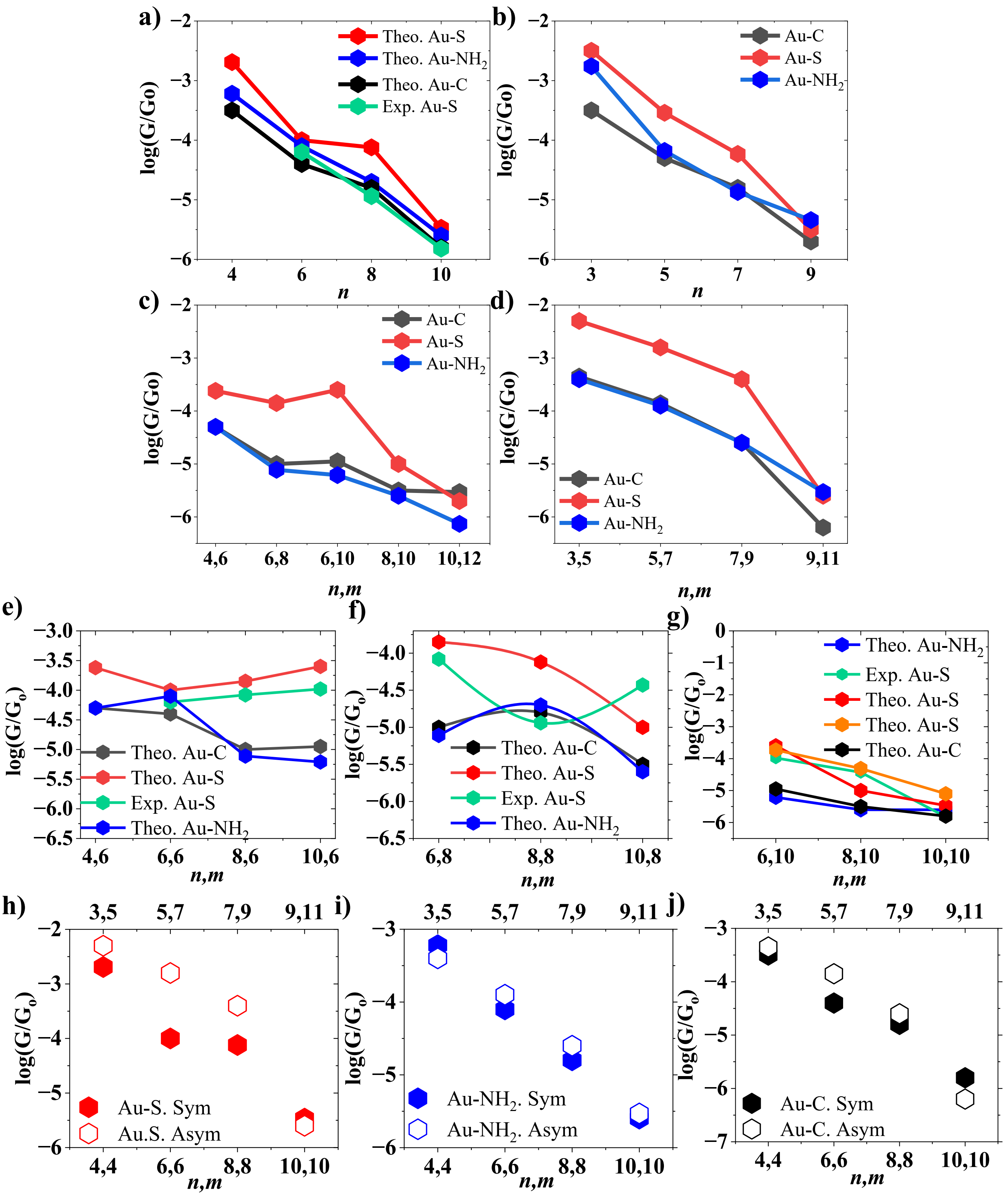

Figure S16. Length-dependent conductance of Au|cyclic molecule|Au single-molecule junctions with different molecular symmetries and anchoring groups. **(a–d)** Show the density functional theory (DFT)–calculated conductance as a function of molecular length for cyclic alkane molecules with different branch parity and symmetry: **(a)** symmetric cyclic molecules with even-numbered branches, **(b)** symmetric cyclic molecules with odd-numbered branches, **(c)** asymmetric cyclic molecules with even-numbered branches, and **(d)** asymmetric cyclic molecules with odd-numbered branches. In each case, conductance values are reported for three anchoring groups: thiol (Au–S, red), amine (Au–NH_2, blue), and direct carbon contact (Au–C, black). Experimental scanning tunneling microscopy (STM) conductance data for thiol-terminated junctions are included, where available (green), for comparison

[18]. **(e–g)** Compare theoretical and experimental logarithmic conductance values as a function of the total number of carbon atoms for cyclic molecules with fixed branch lengths: **(e)** 6-unit branches, **(f)** 8-unit branches , and **(g)** 10-unit branches. DFT results for thiol, amine, and direct carbon anchoring are shown as red, blue, and black hexagons, respectively. Experimental STM data for thiol-anchored junctions (green hexagons) and previously reported theoretical results (orange hexagons) are also included for comparison [18,19]. **(h–j)** Summary of logarithmic electrical conductance for symmetric and asymmetric cyclic alkane molecules featuring a constant upper carbon branch and a variable lower branch with three different anchor groups: **(h)** thiol (–SH), **(i)** amine (–NH_2), and **(j)** direct carbon (–C) anchoring groups. Symmetric cyclic molecules (lower x-axis) and asymmetric cyclic molecules (upper x-axis) are distinguished by different hexagon colours. For all anchoring groups, symmetric and asymmetric molecules with identical carbon counts exhibit similar conductance values.

S7. Individual Conductance Plots

In this section, we present the original individual figures that have been combined into the multi-panel Figure 1 in the main manuscript. Each figure highlights a specific aspect of the length-dependent conductance behavior of cyclic molecular junctions terminated with three different anchoring groups. For clarity and completeness, the individual figures corresponding to Figures 1–6 in the original presentation are provided here, allowing the reader to examine the detailed trends and comparisons discussed in the main text.

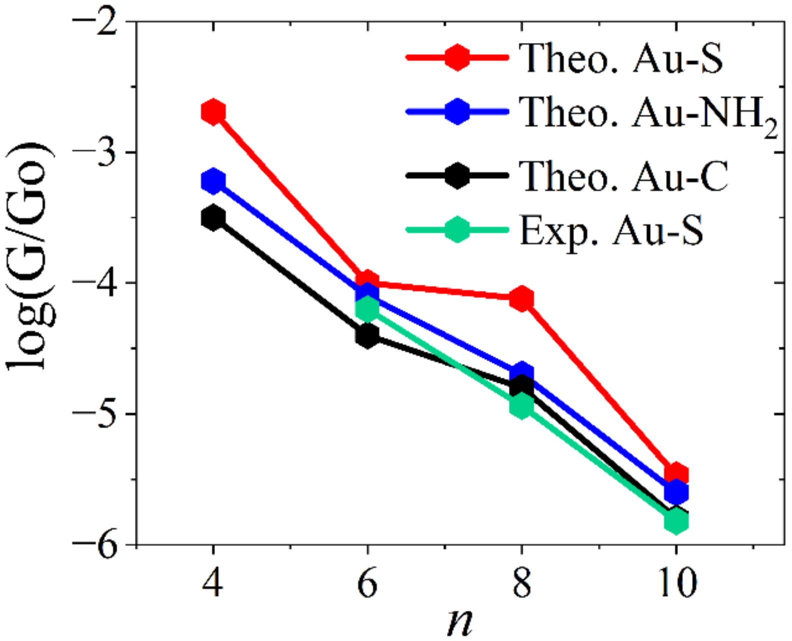

Figure S17. Shows the length-dependent conductance of Au|cyclic molecule|Au junctions with three different terminal groups. The DFT-calculated conductance of symmetric cyclic molecules with even-numbered branches is plotted as a function of length for three linkers: Au-S (red line), Au-NH2 (blue line), and Au-C (black line). The green line represents the conductance values for Au–S obtained using STM measurements [18].

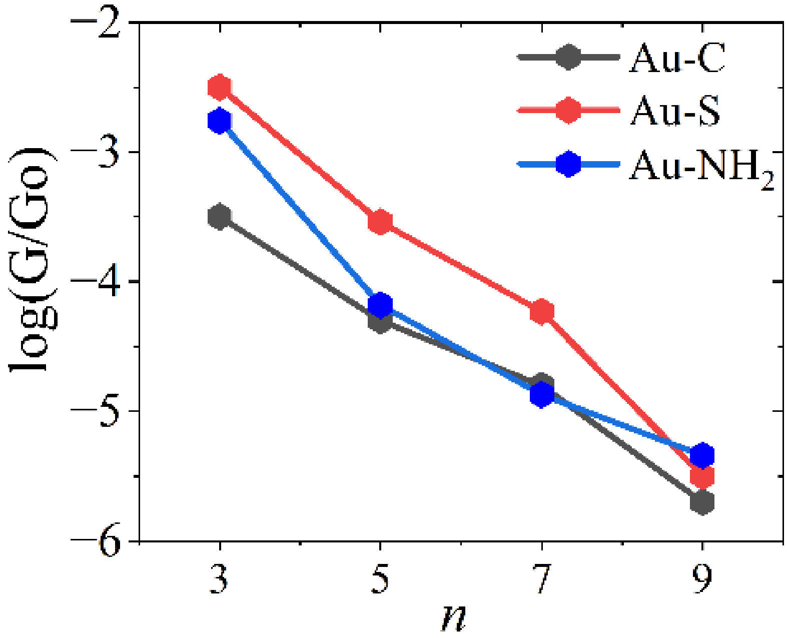

Figure S18. DFT-calculated conductance vs. molecular length for symmetric cyclic molecules with odd-numbered branches. The conductance for three anchoring groups—thiol (Au–S, red), amine (Au–NH2, blue), and direct carbon (Au–C, black)—in a gold–molecule–gold junction demonstrates classical length-dependent decay, with Au–S providing the highest conductance.

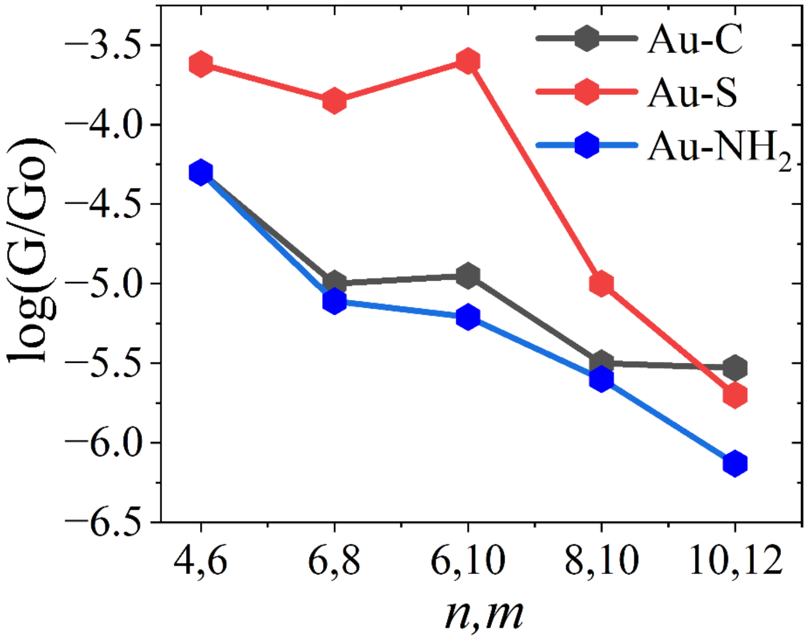

Figure S19. Length dependence of the conductance for Au|cyclic molecule|Au junctions. Density functional theory (DFT)-calculated conductance of even-numbered asymmetric cyclic molecules is shown as a function of molecular length (or electrode separation) for three different anchor groups: thiol (Au–S, red), amine (Au–NH2, blue), and direct carbon (Au–C, black).

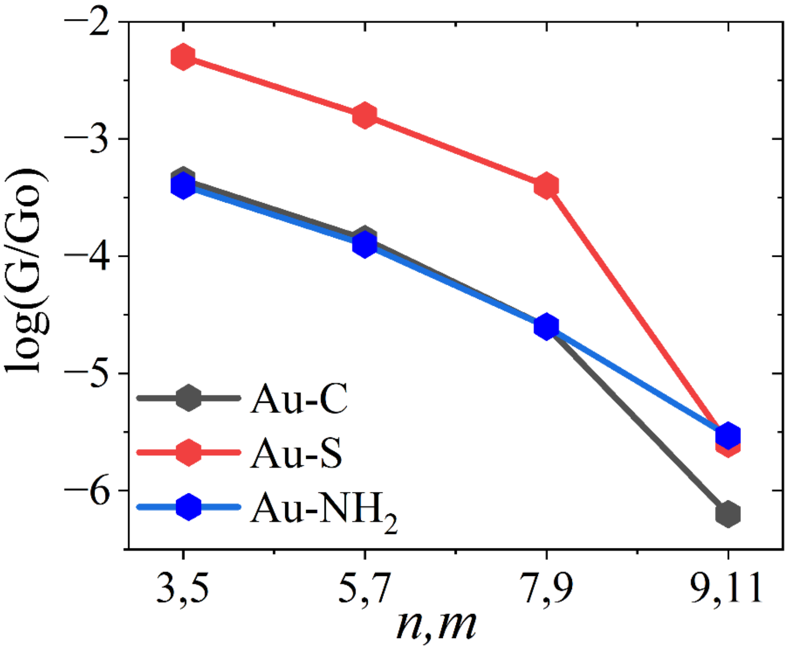

Figure S20. Length-dependent conductance of asymmetric cyclic molecules. Calculated DFT conductance of odd-numbered asymmetric alkane cyclic molecules in Au|molecule|Au junction, shown as a function of molecular length for three different anchor groups: Au–C (black), Au–S (red), and Au–NH2 (blue).

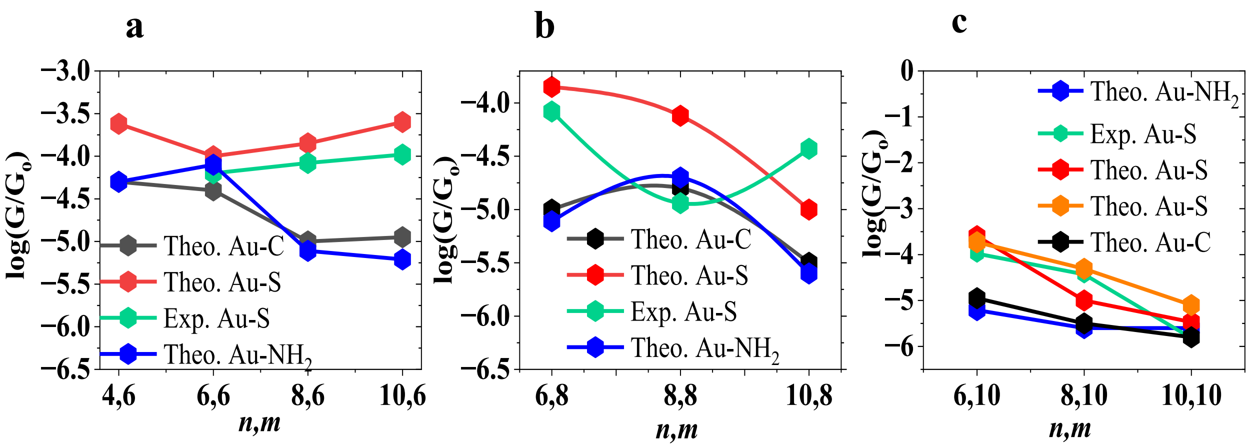

Figure S21. Theoretical and exper imental conductance of Au|cyclic molecule|Au junctions. The logarithmic conductance is shown as a function of the total number of carbon atoms for symmetric and asymmetric alkane cyclic molecules with three different terminal groups: thiol (−SH), amine (−NH2), and direct carbon (−C) contact. **(a)** represents the logarithmic conductance of the 6-unit branch (common C6 branch) for three different anchor groups, while **(b)** and **(c)** present the logarithmic conductance of the 8-unit and 10-unit branches, respectively, using the same set of anchors.Theoretical data from DFT calculations for these groups are represented by red, blue, and black hexagons, respectively. Experimental data [18] includes STM conductance values with thiol anchors (green hexagons) and data from a prior theoretical study [19] (orange hexagons).

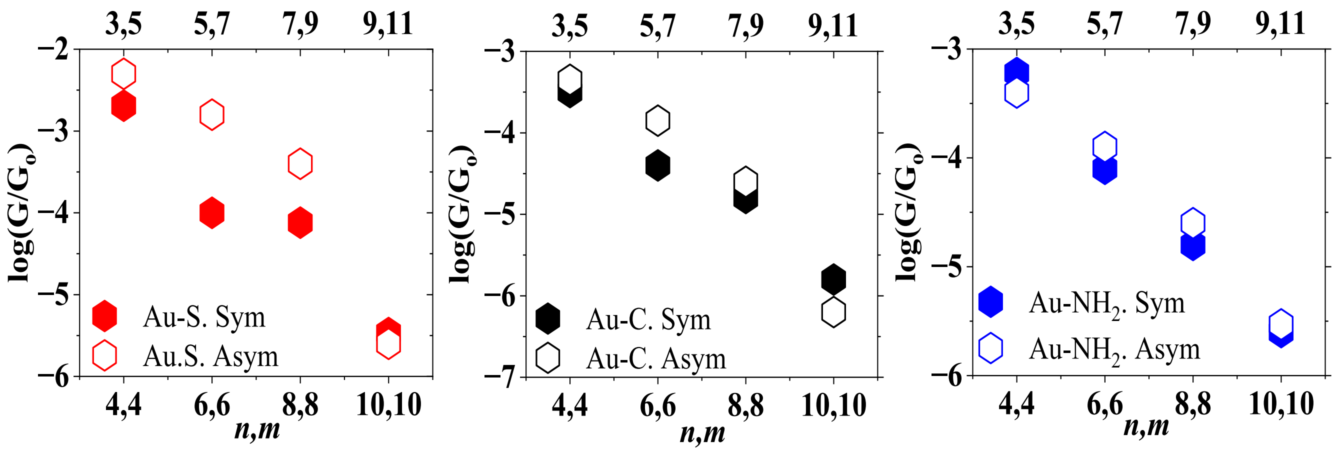

Figure S22. Theoretical logarithmic conductance of single-molecule junctions with symmetric and asymmetric alkane cyclic molecules. The length dependence of the conductance for Au|molecule|Au junctions is shown for symmetric and asymmetric alkane cyclic molecules. **(a)** represents logarithmic conductance with the thiol anchor (−SH), while **(b)** and **(c)** show logarithmic conductance with the amine (−NH_2) and direct carbon (−C) anchor groups, respectively. Density Functional Theory (DFT) calculations predict that conductance decreases with increasing molecular size (total number of carbon atoms) for all terminal groups. Asymmetric cyclic molecules (upper X-axis) and symmetric cyclic molecules (bottom X-axis) are represented by different symbols: red, blue, and black hexagons for symmetric molecules with thiol, amine, and direct carbon terminals, respectively, and red, blue, and black empty hexagons for their asymmetric counterparts

References

1. Kobko, N., & Dannenberg, J. J. (2001). Effect of Basis Set Superposition Error (BSSE) upon ab Initio Calculations of Organic Transition States. *The Journal of Physical Chemistry A*, *105*(10), 1944–1950.

2. Kohn, W., & Sham, L. J. (1965). Self-Consistent Equations Including Exchange and Correlation Effects. *Physical Review*, *140*(4A), A1133–A1138.

3. Perdew, J. P., Burke, K., & Ernzerhof, M. (1996). Generalized Gradient Approximation Made Simple. *Physical Review Letters*, *77*(18), 3865–3868.

4. Perdew, J. P., & Zunger, A. (1981). Self-interaction correction to density-functional approximations for many-electron systems. *Physical Review. B, Condensed Matter*, *23*(10), 5048–5079.

5. Sinnokrot, M. O., Valeev, E. F., & Sherrill, C. D. (2002). Estimates of the Ab Initio Limit for π−π Interactions: The Benzene Dimer. *Journal of the American Chemical Society*, *124*(36), 10887–10893.

6. Soler, J. M., Artacho, E., Gale, J. D., García, A., Junquera, J., Ordejón, P., & Sánchez-Portal, D. (2002). The SIESTA method forab initioorder-Nmaterials simulation. *Journal of Physics Condensed Matter*, *14*(11), 2745–2779.

7. Cao, Y., Huang, C., & Lu, Q. (2024). Photoelectrochemically driven iron-catalysed C(sp3)−H borylation of alkanes. *Nature Synthesis*, *3*(4), 537–544.

8. Ding, W. W., He, Z. Y., Sayed, M., Zhou, Y., Han, Z. Y., & Gong, L. Z. (2024). Enantioselective synthesis of β- and α-amino ketones through reversible alkane carbonylation. *Nature Synthesis*, *3*(4), 507–516**.**

9. Dragojlovic, V. Conformational analysis of cyclics. *ChemTexts* **1**, 14 (2015).

10. Ahluwalia, V., & Aggarwal, R. (2023). *Alicyclic Chemistry*. Springer Nature.

11. Chickos, J. S., Hesse, D. G., Panshin, S. Y., Rogers, D. W., Saunders, M., Uffer, P. M., & Liebman, J. F. (1992). The strain energy of cyclotetradecane is small. *Journal of Organic Chemistry*, *57*(6), 1897–1899.

12. Meng, X., Lu, H., Zhang, Z., Peng, P., & Volkman, J. K. (2023). Structural characterization and mass spectrometry fragmentation signatures of macrocyclic alkanes isolated from a Sydney Basin torbanite, Australia. *Acta Geochimica,* 42(3), 488–494**.**

13. Swain, S., Bej, S., Bishoyi, A.K. *et al.* Recent progression on phytochemicals and pharmacological properties of the filamentous cyanobacterium *Lyngbya* sp. *Naunyn-Schmiedeberg's Arch Pharmacol* **396**, 2197–2216 (2023).
14. Zhang, S., Wang, X., Su, Y., Qiu, Y., Zhang, Z., & Wang, X. (2014). Isolation and reversible dimerization of a selenium–selenium three-electron σ-bond. *Nature Communications*, *5*(1).

15. National Center for Biotechnology Information (2024). PubChem Compound Summary for CID 12560936, 1,7-Dithiacyclododecane. Retrieved July 9, 2024 from https://pubchem.ncbi.nlm.nih.gov/compound/1_7-Dithiacyclododecane.

16. Müller, A., Funder-Fritzsche, E., Konar, W. *et al.* Thia- und Dithia-cyclic und die Spaltung einiger gesättigter Heterocyclen mit Methyljodid. *Monatshefte für Chemie* **84**, 1206–1220 (1953).

17. Glass, R. S. (1990). Design, Synthesis, and Conformational Analysis of Compounds Tailored for the Study of Sulfur-Centered Reactive Intermediates. In *Springer eBooks* (pp. 227–238).

18. Müller, A., Funder-Fritzsche, E., Konar, W. *et al.* Thia- und Dithia-cyclic und die Spaltung einiger gesättigter Heterocyclen mit Methyljodid. *Monatshefte für Chemie* **84**, 1206–1220 (1953).

19. Ye, J., Al-Jobory, A., Zhang, Q. C., Cao, W., Alshehab, A., Qu, K., Alotaibi, T., Chen, H., Liu, J., Ismael, A. K., Chen, Z. N., Lambert, C. J., & Hong, W. (2022). Highly insulating alkane rings with destructive σ-interference. *Science China Chemistry*, *65*(9), 1822–1828.

20. National Center for Biotechnology Information (2024). PubChem Compound Summary for CID 544123, 1,6-Dithiacyclododecane. Retrieved July 9, 2024 from https://pubchem.ncbi.nlm.nih.gov/compound/1_6-Dithiacyclododecane.

21. Cheng, Y., & Qin, D. (2024). *Classification of Diverse Novel Alkaloids* (pp. 117–149).

22. National Center for Biotechnology Information (2024). PubChem Compound Summary for CID 14290132, 1,7-Diazacyclododecane. Retrieved September 2, 2024 from https://pubchem.ncbi.nlm.nih.gov/compound/1_7-Diazacyclododecane.

23. National Center for Biotechnology Information (2024). PubChem Compound Summary for CID 19890075, 1,9-Diazacyclohexadecane. Retrieved September 2, 2024 from https://pubchem.ncbi.nlm.nih.gov/compound/1_9-Diazacyclohexadecane.

24. Müller, A., Šrepel, E., Funder-Fritzsche, E. *et al.* Aza- und Diaza-cyclic. *Monatshefte für Chemie* **83**, 386–393 (1952).

25. Alder, R. W. (2005). Design of C2-Chiral Diamines That Are Computationally Predicted To Be a Million-fold More Basic than the Original Proton Sponges. *Journal of the American Chemical Society*, *127*(21), 7924–7931.

26. Mikhura, I.V., Formanovskii, A.A. Synthesis of aza-crown compounds by intramolecular cyclization of ω-amino acids. *Chem Heterocycl Compd* **28**, 205–212 (1992).

27. Müller, A., Funder-Fritzsche, E., Konar, W. *et al.* Thia- und Dithia-cyclic und die Spaltung einiger gesättigter Heterocyclen mit Methyljodid. *Monatshefte für Chemie* **84**, 1206–1220 (1953).

28. National Center for Biotechnology Information (2024). PubChem Compound Summary for CID 154128457, 1,12-Diazacyclodocosane. Retrieved September 2, 2024 from https://pubchem.ncbi.nlm.nih.gov/compound/1_12-Diazacyclodocosane.

29. National Center for Biotechnology Information (2024). PubChem Compound Summary for CID 12793068, 1,5-Diazecane. Retrieved September 3, 2024 from https://pubchem.ncbi.nlm.nih.gov/compound/1_5-Diazecane.

30. National Center for Biotechnology Information (2024). PubChem Compound Summary for CID 70649771, 1,7-Diazacyclotetradecane. Retrieved September 3, 2024 from https://pubchem.ncbi.nlm.nih.gov/compound/1_7-Diazacyclotetradecane.

31. National Center for Biotechnology Information (2024). PubChem Compound Summary for CID 122550382, 1,6-Diazacyclododecane. Retrieved September 3, 2024 from https://pubchem.ncbi.nlm.nih.gov/compound/1_6-Diazacyclododecane.

32. National Center for Biotechnology Information (2024). PubChem Compound Summary for CID 13128616, 1,8-Diazacyclooctadecane. Retrieved September 3, 2024 from https://pubchem.ncbi.nlm.nih.gov/compound/1_8-Diazacyclooctadecane.

33. Venkataraman, L., Klare, J. E., Nuckolls, C., Hybertsen, M. S., & Steigerwald, M. L. (2006). Dependence of single-molecule junction conductance on molecular conformation. *Nature*, **442**(7105), 904–907.

34. Chen, F., Hihath, J., Huang, Z., Li, X., & Tao, N. (2006). Measurement of single-molecule conductance. *Nano Letters*, **6**(7), 1589–1594.

35. Quek, S. Y., Kamenetska, M., Steigerwald, M. L., Choi, H. J., Louie, S. G., Nuckolls, C., Hybertsen, M. S., Neaton, J. B., & Venkataraman, L. (2007). Mechanically controlled binary conductance switching of a single-molecule junction. *Nano Letters*, **7**(11), 3477–3482.

36. Chen, F., Li, X. L., Hihath, J., Huang, Z. F., & Tao, N. J. (2006). Effect of anchoring groups on single-molecule conductance: Comparative study of thiol-, amine-, and carboxylic-acid-terminated molecules. *Journal of the American Chemical Society*, **128**(49), 15874–15881.
